# Scaling Up the 2010 World Health Organization HIV Treatment Guidelines in Resource-Limited Settings: A Model-Based Analysis

**DOI:** 10.1371/journal.pmed.1000382

**Published:** 2010-12-21

**Authors:** Rochelle P. Walensky, Robin Wood, Andrea L. Ciaranello, A. David Paltiel, Sarah B. Lorenzana, Xavier Anglaret, Adam W. Stoler, Kenneth A. Freedberg, for the CEPAC-International Investigators

**Affiliations:** 1 Division of Infectious Disease, Department of Medicine, Massachusetts General Hospital, Boston, Massachusetts, United States of America; 2 Division of General Medicine, Department of Medicine, Massachusetts General Hospital, Boston, Massachusetts, United States of America; 3 Division of Infectious Disease, Brigham and Women's Hospital, Boston, Massachusetts, United States of America; 4 Harvard University Center for AIDS Research, Harvard Institute for Global Health, Cambridge, Massachusetts, United States of America; 5 Department of Health Policy and Management, Harvard School of Public Health, Boston, Massachusetts, United States of America; 6 Desmond Tutu HIV Centre, Institute of Infectious Disease and Molecular Medicine, University of Cape Town, Cape Town, South Africa; 7 Yale School of Medicine, New Haven, Connecticut, United States of America; 8 INSERM Unité 897, Centre de Recherche “Epidémiologie et Biostatistique”, Bordeaux Cedex, France; 9 Université Victor Segalen Bordeaux 2, Bordeaux, France; University of Berne, Switzerland

## Abstract

**Background:**

The new 2010 World Health Organization (WHO) HIV treatment guidelines recommend earlier antiretroviral therapy (ART) initiation (CD4<350 cells/µl instead of CD4<200 cells/µl), multiple sequential ART regimens, and replacement of first-line stavudine with tenofovir. This paper considers what to do first in resource-limited settings where immediate implementation of all of the WHO recommendations is not feasible.

**Methods and Findings:**

We use a mathematical model and local input data to project clinical and economic outcomes in a South African HIV-infected cohort (mean age = 32.8 y, mean CD4 = 375/µl). For the reference strategy, we assume that all patients initiate stavudine-based ART with WHO stage III/IV disease and receive one line of ART (stavudine/WHO/one-line). We rank—in survival, cost-effectiveness, and equity terms—all 12 possible combinations of the following: (1) stavudine replacement with tenofovir, (2) ART initiation (by WHO stage, CD4<200 cells/µl, or CD4<350 cells/µl), and (3) one or two regimens, or lines, of available ART. Projected life expectancy for the reference strategy is 99.0 mo. Considering each of the guideline components separately, 5-y survival is maximized with ART initiation at CD4<350 cells/µl (stavudine/<350/µl/one-line, 87% survival) compared with stavudine/WHO/two-lines (66%) and tenofovir/WHO/one-line (66%). The greatest life expectancies are achieved via the following stepwise programmatic additions: stavudine/<350/µl/one-line (124.3 mo), stavudine/<350/µl/two-lines (177.6 mo), and tenofovir/<350/µl/two-lines (193.6 mo). Three program combinations are economically efficient: stavudine/<350/µl/one-line (cost-effectiveness ratio, US$610/years of life saved [YLS]), tenofovir/<350/µl/one-line (US$1,140/YLS), and tenofovir/<350/µl/two-lines (US$2,370/YLS).

**Conclusions:**

In settings where immediate implementation of all of the new WHO treatment guidelines is not feasible, ART initiation at CD4<350 cells/µl provides the greatest short- and long-term survival advantage and is highly cost-effective.

*Please see later in the article for the Editors' Summary*

## Abstract 2

Rochelle Walensky and colleagues use a model-based analysis to examine which of the 2010 WHO antiretroviral therapy guidelines should be implemented first in resource-limited settings by ranking them according to survival, cost-effectiveness, and equity.

## Editors' Summary

**Background:**

Since 1981, acquired immunodeficiency syndrome (AIDS) has killed more than 25 million people, and about 33 million people (30 million of them in low- and middle-income countries) are now infected with the human immunodeficiency virus (HIV), which causes AIDS. HIV destroys immune system cells (including CD4 cells, a type of lymphocyte), leaving infected individuals susceptible to other infections (so-called opportunistic infections). Early in the AIDS epidemic, most people with HIV died within 10 years of infection. Then, in 1996, highly active antiretroviral therapy (ART)—a combination of several powerful antiretroviral drugs—was developed. Now, in resource-rich countries, clinicians care for people with HIV by prescribing ART regimens tailored to each individual's needs. They also regularly measure the amount of virus in their patients' blood, test for antiretroviral-resistant viruses, and monitor the health of their patients' immune systems through regular CD4 cell counts. As a result, the life expectancy of patients with HIV in developed countries has dramatically improved.

**Why Was This Study Done?:**

Initially, resource-limited countries could not afford to provide ART for their populations, and the life expectancy of HIV-positive people remained low. Now, through the concerted efforts of governments, the World Health Organization (WHO), and other international agencies, more than a third of the people in low- and middle-income countries who need ART are receiving it. However, many without access are still in need of ART, and ART programs in developing countries follow a public-health approach rather than an individualized approach. That is, drug regimens, clinical decision-making, and disease monitoring are all standardized and follow recommendations in the 2006 WHO ART guidelines. This year (2010), these guidelines were revised. The guidelines now recommend the following: earlier ART initiation—when the CD4 count falls below 350/µl of blood, instead of below 200/µl as in the 2006 guidelines; the provision of sequential ART regimens instead of a single regimen; and the replacement of the antiretroviral drug stavudine with tenofovir, a less toxic but more expensive drug, in first-line ART regimens. However, many resource-limited countries are still struggling to implement the 2006 guidelines, so which of these new recommendations should be prioritized? Here, the researchers use a mathematical model to address this question.

**What Did the Researchers Do and Find?:**

The Cost Effectiveness of AIDS Complications (CEPAC)–International model simulates the natural history and treatment of HIV disease. The researchers entered South African clinical and cost data for HIV treatment into this model and then used it to project survival and costs in a hypothetical group of South African HIV-positive patients under alternative guideline prioritization scenarios. The reference strategy for the simulations (denoted as “stavudine/WHO/one-line”) assumed that patients (with a mean CD4 count of 375/µl) began a single stavudine-based ART regimen when they developed WHO stage III/IV HIV disease (i.e., when patients develop multiple opportunistic infections such as tuberculosis and pneumonia). When the new guideline recommendations were considered separately, ART initiation at CD4<350/µl (stavudine/<350/µl/one-line) maximized five-year survival. Stepwise adjustment from the reference strategy (which had a life expectancy 99.0 months) through strategies of stavudine/<350/µl/one-line (a projected life expectancy of 124.3 months), stavudine/<350/µl/two-lines (177.6 months), and tenofovir/<350/µl/two-lines (193.6 months) produced the greatest improvements in life expectancy. Finally, strategies of stavudine/<350/µl/one-line, tenofovir/<350/µl/one-line, and tenofovir/<350µl/two-lines produced incremental cost-effectiveness ratios of US$610, US$1,140, and US$2,370 per year of life saved, respectively.

**What Do These Findings Mean?:**

As with all mathematical models, the accuracy of these findings are dependent on the assumptions included in the model and on the data populating it. Nevertheless, these findings suggest that, where resources are limited and immediate implementation of all the new WHO recommendations is impossible, ART initiation at a CD4 count of less than 350/µl would provide the greatest survival advantage and would be very cost-effective. In countries that are already initiating ART at this threshold and that have access to CD4 monitoring, a switch from stavudine to tenofovir would further increase survival and would also be cost-effective. Finally, although access to second-line ART regimen would provide more clinical benefits than access to tenofovir, the cost of this change in strategy would be substantially greater. Importantly, these findings should help to avoid the complete dismissal of the revised WHO guidelines on the basis of cost and should help policy makers adjust their ART program strategies to maximize their clinical benefits and cost effectiveness.

**Additional Information:**

Please access these Web sites via the online version of this summary at http://dx.doi.org/10.1371/journal.pmed.1000382.

- Information is available from the US National Institute of Allergy and Infectious Diseases on HIV infection and AIDS
- HIV InSite has comprehensive information on all aspects of HIV/AIDS
- Information is available from Avert, an international AIDS charity, on many aspects of HIV/AIDS, including information on HIV/AIDS in South Africa and on HIV/AIDS treatment and care (in English and Spanish)
- WHO provides information about universal access to AIDS treatment (in English, French, and Spanish); its 2010 ART guidelines can be downloaded
- More information on the CEPAC model is available

## Introduction

The 2006 World Health Organization (WHO) guidelines on antiretroviral therapy (ART) established a worldwide standard of care for patients with HIV infection [1]. Since this publication, new evidence has emerged on how to treat patients infected with HIV, and this evidence formed the basis for the WHO 2010 ART guidelines update [2]. These revisions aim to better align global standards with those already adopted in well-resourced countries [3],[4]. Specifically, revised guidelines recommend an increased number of sequential ART regimens, routinely available CD4 count monitoring, earlier ART initiation thresholds (CD4<350 cells/µl versus CD4<200 cells/µl), and replacement of stavudine with the less-toxic drug tenofovir.

As WHO expands treatment recommendations, many countries in resource-limited settings still struggle to implement 2006 guidelines [5]. In Malawi, for example, most HIV disease is monitored clinically; CD4 count monitoring is limited to pregnant women and children [6],[7]. In South Africa, ART is available to only 22%–36% of those reported to be in need [8]. In settings confronted with numerous new recommendations, not all of which are immediately feasible, the relevant policy question is: *What to do first?* Should countries begin by replacing stavudine with tenofovir or by making CD4 count monitoring universally available? To assist policy makers in this prioritization process, we use a model-based analysis with data from South Africa to project the clinical and economic outcomes of alternative stepwise implementation scenarios toward the 2010 WHO ART guidelines.

## Methods

### Analytic Overview

**Figure 1:**
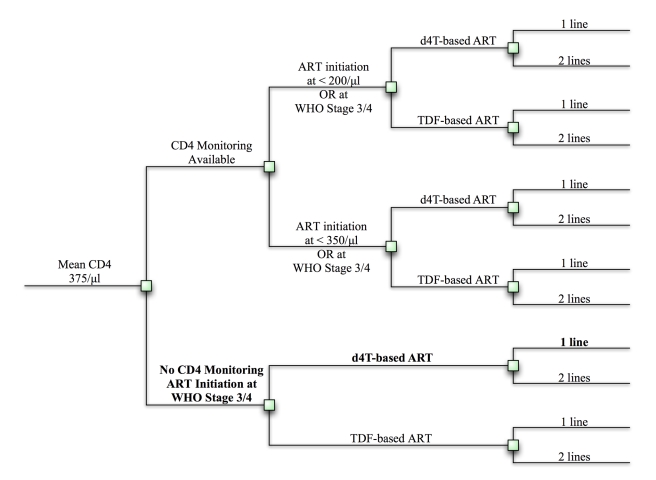
Clinical and policy decisions yield 12 implementation strategies. Clinical and policy decisions result in 12 possible implementation strategies. These strategies are listed in Text S1. Squares represent decision points. The reference strategy is bolded. d4t, stavudine; TDF, tenofovir.

The Cost Effectiveness of AIDS Complications (CEPAC)–International model is a Monte Carlo simulation model of the natural history and treatment of HIV disease (see Text S1 for model details) [9]–[11]. We populate the model with South African clinical and resource utilization data to project survival and costs under alternative guideline prioritization scenarios. We use a “no ART” scenario for comparison and assume that baseline care (designated the “reference strategy”) is a one-line stavudine-containing regimen, initiated at WHO stage III or IV disease, without CD4 count monitoring capacity. We then examine every feasible sequence of the following implementation elements: (1) widespread CD4 count monitoring capacity, allowing for ART initiation at CD4<200 cells/µl (and biannual monitoring), (2) earlier ART initiation, at CD4<350 cells/µl (assumes CD4 count availability), (3) an available second-line ART regimen upon first-line failure, and (4) replacement of stavudine with tenofovir in the first-line regimen. To refer to these strategies, we use the following nomenclature: nucleoside analog in first line/ART initiation criterion/number of regimens [e.g., stavudine/<200/µl/two-lines]). The combined implementation elements result in twelve possible strategies, in addition to no ART (Figures 1 and S1). We examine the short- and long-term survival benefits and cost-effectiveness of each stepwise, incremental policy change from the reference strategy to full 2010 guideline implementation. We also evaluate the cost and survival impact of imposing an additional “equity” constraint—i.e., that all members of the cohort at any given time are provided the same treatment program. Finally, we use sensitivity analyses to examine the efficacy and cost input parameters necessary to change the conclusions.

When reporting clinical outcomes alone (per-person life expectancy), we provide undiscounted results. When clinical and economic results are used to create cost-effectiveness ratios, we adhere to established convention in discounting both at 3% per annum [12]; cost-effectiveness ratios are reported in US dollars per year of life saved (dollars/YLS) (See Text S1 for details). We conduct an “incremental” assessment of economic costs and health benefits, as recommended by the US Panel on Cost-Effectiveness in Health and Medicine [12]. Cost and health outcomes are estimated for all 12 strategies (as well as no ART). These are then ranked in order of increasing cost. After eliminating all “dominated” strategies (i.e., strategies that both cost more and confer fewer benefits than any combination of other strategies), we compute the ratio of incremental costs to incremental benefits for each strategy, comparing it to its next-least-costly, non-dominated alternative [12].

Costs are converted to 2008 US dollars using the South African gross domestic product deflators and the 2008 mean exchange rate between the South African rand and the US dollar (8.23 rand = US$1) [13],[14]. Guided by the recommendations of the WHO Commission on Macroeconomics and Health, we consider interventions to be cost-effective in a given country if their cost-effectiveness ratio is less than 3 times the national per capita gross domestic product (South African 2008 gross domestic product = US$5,700) [14].

### The CEPAC-International Model

The CEPAC-International model simulates the progression of disease in a hypothetical cohort of patients infected with HIV as a sequence of monthly transitions between health states. Health states are defined to be clinically and economically representative of the disease course and are stratified by current CD4 count, current HIV RNA level, and history of opportunistic disease. A graphical representation of a patient trace in South Africa is presented in Figure S2, illustrating CD4 cell count, HIV RNA, and clinical events, including tuberculosis, over a hypothetical patient's lifetime. We are careful to distinguish in the model “actual” CD4 cell count and HIV RNA—i.e., the underlying immunologic and virologic state, regardless of whether they are measured by a laboratory test—from “observed” CD4 cell count and HIV RNA—that which is measured by a test and upon which clinical decisions can be made. Actual CD4 cell count determines the frequency of opportunistic diseases, while ART influences actual HIV RNA levels and CD4 cell counts. Health states therefore reflect the underlying disease process, and clinical decisions (ART initiation or switch) are based on observed factors such as presentation with an opportunistic disease, or CD4 count, if monitoring is available. Reflecting standards of care in most sub-Saharan African nations, HIV RNA monitoring is assumed to be unavailable [1]. Patients are followed from entry into HIV care through death.

In strategies without available CD4 monitoring, decisions regarding ART initiation and switching are made based upon observation of any of the following severe opportunistic diseases representative of WHO stage III/IV disease: severe bacterial infection, severe fungal infection, tuberculosis, toxoplasmosis, nontuberculous mycobacteriosis, *Pneumocystis jiroveci* pneumonia, or other WHO stage IV defining diseases. Two mild opportunistic diseases (fungal and other) result in resource utilization but no changes in the ART decision-making process. Patients die in the model from an acute event (e.g., an opportunistic disease or a drug-related toxicity), from chronic HIV disease, or at South African age- and sex-adjusted background mortality rates [15].

The frequency of clinical and laboratory assessments in the model is user-defined. For this analysis, we have chosen clinical assessments to occur every 3 mo; in strategies where CD4 counts are available, they are modeled as being performed biannually. ART is initiated when one of two criteria is met: falling below a defined CD4 count threshold or the development of WHO III/IV disease (i.e., severe opportunistic disease). Effective ART in the model results in actual virologic suppression (independent of gender), a CD4 count increase, and a CD4-independent reduction in risk of opportunistic diseases and chronic AIDS death [16]–[19]. Because HIV RNA monitoring is unavailable, virologic failure on an antiretroviral regimen is itself not detected. However, the impact of virologic failure is ultimately observed when it manifests with immunologic dysfunction through either a documented opportunistic disease or a CD4 decline that is revealed by laboratory testing. Six months after ART initiation, observed treatment failure is defined as meeting any one of the following three criteria: the development of a severe opportunistic disease, observation of a 50% decline from peak on-treatment CD4 count, or observation of two consecutive CD4 counts below 100 cells/µl [1]. Upon observed treatment failure, ART is switched if a subsequent regimen is available or, if not, the failed regimen is continued until death to maintain its modest decreases in the rates of opportunistic disease and death [17],[18]. For the purposes of this analysis, we assume no treatment interruptions.

Stavudine- or tenofovir-related toxicity occurs with a one-time probability, distributed over time since drug initiation. Depending on the nature of the toxicity, toxicity results in a one-time cost and/or a duration of costs spanning the time of increased need for care. Certain types of toxicity—including lactic acidosis, lipodystropy, neuropathy, and nephrotoxicity—also result in a single drug switch to zidovudine.

### Evaluating Uncertainty

To converge on stable model output, we run a simulated cohort of 1 million patients infected with HIV. Because the cohort size can be varied in the simulation—i.e., we might also simulate 2 million or 5 million patients—95% confidence intervals and standard deviations (SDs) do not adequately capture uncertainty in simulation modeling. Instead, we adhere to the guidance of the US Panel on Cost-Effectiveness in Health and Medicine for reporting uncertainty in deterministic methods [12]. We use univariate sensitivity analysis to examine the impact of variation in individual input parameters. Having identified those variables that exert the greatest influence on our conclusions, we then turn to multivariate sensitivity analyses to examine the impact of simultaneous variation in multiple parameters. This approach results in a large variety of univariate and multivariate sensitivity analyses. We report those instances in which variation of an underlying parameter value has material impact on the findings and conclusions. A more comprehensive description of relevant sensitivity analyses is provided in Texts S2 and S3.

### Input Parameters

Data sources for individual input parameters are referenced in Table 1 and in Text S1.

**Table 1:** Model input parameters for analysis of the 2010 WHO ART guidelines.

Variable	Estimate	Reference
**Initial cohort characteristics**		
Age, mean years ± SD	32.8±9.2	[20]
Male (%)	54.6	[20]
Distribution of initial CD4, mean cells/µl (SD)	375 (25)	Assumption
HIV RNA distribution (%)		[21]
>100,000 copies/ml	42.5	
30,001–100,000 copies/ml	28.3	
10,001–30,000 copies/ml	17.9	
3,001–10,000 copies/ml	7.8	
501–3,000 copies/ml	2.3	
<500 copies/ml	1.2	
**Natural history of disease**		
Mean monthly CD4 decline (cells/µl) by HIV RNA stratum (copies/ml)		[35]
>30,000	6.4	
10,001–30,000	5.4	
3,001–10,000	4.6	
501–3,000	3.7	
Monthly risk of severe opportunistic infections (%)^a^		[20]
Bacterial	0.08–0.71	
Fungal	0.02–2.22	
Tuberculosis	0.21–1.96	
Toxoplasmosis	0.00–0.06	
Nontuberculosis mycobacteriosis	0.00–0.30	
*P. jiroveci* pneumonia	0.00–0.12	
Other severe opportunistic infections	0.25–2.57	
Monthly risk of mild opportunistic diseases (%)^a^		[20]
Fungal	0.59–3.51	
Other	2.51–3.10	
**Efficacy of co-trimoxazole (% reduction in probability of infection)**		
Severe bacteria	49.8	[22],[23]
Mild fungal infections	−46.4^b^	[22],[23]
Toxoplasmosis	83.2	[22],[23]
*P. jiroveci* pneumonia	97.3	[22],[23]
Other WHO stage IV defining diseases	17.9	[22]
**Efficacy of ART (range examined)**		
First line: stavudine-based regimen		[19]
HIV RNA suppression	75% at 24 wk	
CD4 count increase	136 cells/µl at 48 wk	
Probability of later failure (monthly, after 24 wk)	0.02^c^ (0.01–0.02)	
First line: tenofovir-based regimen		
HIV RNA suppression	85% at 24 wk (85%–95%)	[24]
CD4 count increase	136 cells/µl at 48 wk	[19]
Probability of later failure (monthly, after 24 wk)	0.01^d^ (0.005–0.01)	[24]
Second line: lopinavir/ritonavir-based regimen		[16]
HIV RNA suppression	78% at 24 wk (40%–88%)	
CD4 count increase	151 cells/µl at 48 wk	
Probability of later failure (monthly, after 48 wk)	0.03^e^ (0.01–0.06)	
**Toxicity (one-time probability [%])**		
Stavudine-based regimen (range examined)		
Severe lactic acidosis	1.7 (1.7–3.4)	[36]
Lipodystrophy	1.3 (1.3–2.6)	[36]
Neuropathy	2.6 (2.6–5.2)	[36]
Tenofovir-based regimen (range examined)		
Nephrotoxicity	1.6 (1.6–3.2)	[37],[38]
Anemia	0.4 (0.4–0.8)	[24]
**Discount rate**	3%	[12]
**Costs (2008 US dollars) (range examined)**		
Co-trimoxazole prophylaxis (monthly)	1.03	[23]
Stavudine-based first-line ART (monthly)	8.33	[26]
Tenofovir-based first-line ART (monthly)	17.00 (10.00–17.00)	[26]
Lopinavir/ritonavir second-line ART (monthly)	55.75 (8.36–55.75)	[26]
Routine care (range by CD4, monthly)^a^	9.99–131.23	[20],[39],[40]
Inpatient hospital care, per day	224.25	[39]
Outpatient hospital care, per visit	10.87	[39]
CD4 count test	US$25 (25–75)	[27],[28],[41]

“Range examined” indicates that we examined both extreme and intermediate values within the specified ranges.

a Range indicated by CD4 count; details by CD4 strata are presented in the Text S1.

b The percent monthly risk of mild fungal infections is increased by 46.4% in the presence of co-trimoxazole [22].

c Projected using published 24-wk data [19].

d Estimated from published 24- and 48-wk data [24].

e Estimated from published 24- and 48-wk data [16].

#### Cohort characteristics

We define an ART-naïve cohort of patients with HIV in South Africa, with mean age 32.8 y [20]. We intentionally choose an initial cohort with a relatively high mean CD4 cell count of 375 cells/µl (SD, 25 cells/µl). A cohort with a lower mean CD4 cell count would not clearly demonstrate the benefits of an ART initiation threshold of CD4<350 cells/µl, as illustrated in sensitivity analyses (Text S2). Over 40% of the cohort has HIV RNA>100,000 copies/ml (Table 1) [21]. In the model, this ART-naïve cohort is then subject to the policies of ART initiation and drug availability as indicated by each of the 12 strategies. In the absence of ART, the model tracks the patients' natural history of disease for use in comparing the incremental clinical benefits and costs. Figure S3 illustrates the internal validation of South African data used to derive critical model input parameters such as monthly mortality and opportunistic disease incidence rates, stratified by CD4 count.

#### Opportunistic disease prophylaxis and ART efficacy

All patients at model entry are provided co-trimoxazole prophylaxis, conferring protection against mild and severe bacterial infections, *P. jiroveci̧* and toxoplasmosis [22],[23]. We assume a non-nucleoside reverse transcriptase inhibitor–based ART regimen that includes stavudine. This regimen results in a 24-wk virologic suppression rate of 75% with a mean 48-wk CD4 count rise of 136 cells/µl among those with suppression [19]. The monthly probability of virologic failure after 48 wk is 0.02. When stavudine is replaced with tenofovir in first-line regimens, in the absence of reliable efficacy data for a tenofovir-based regimen in resource-limited settings, we use a virologic suppression rate of 85% at 24 wk, as reported in clinical trials [24],[25]. Despite the improved rates of virologic suppression, we want to maintain conservative assumptions with regard to CD4 benefit among those suppressed, so we use the same benefit (136 cells/µl) as that used for the stavudine-based regimen [19]. From these studies, the monthly probability of failure of tenofovir-based ART after 48 wk is 0.01 [24],[25].

When second-line ART is available, it is a lopinavir/ritonavir-based regimen with a 24-wk suppressive efficacy of 78%, a resultant CD4 count increase of 151 cells/µl, and a 0.03 monthly probability of virologic failure after 48 wk [16]. In sensitivity analyses, we examine the impact of improved efficacy of first-line ART associated with the use of tenofovir and the impact of alternative second-line ART efficacies (Text S3).

#### Costs

We consider HIV-associated direct medical costs, including inpatient days, outpatient visits, medication costs, and laboratory tests, when available (Table 1). Direct non-medical costs and indirect costs are excluded. Costs attributable to inpatient hospitalization resulting from an opportunistic infection are calculated as the mean cost of each inpatient day multiplied by the mean length of stay for any given opportunistic disease. Outpatient care costs include the mean cost of each visit, inclusive of standard laboratory tests and procedures. Routine care costs are stratified by CD4 cell count to account for the increased frequency of visits that may be attributable to lower CD4 cell counts (Table 1). The stavudine-based first-line regimen costs US$100 per person-year (stavudine component =  US$36), and the tenofovir-based regimen costs US$204 per person-year (tenofovir component  =  US$135) [26]; all other first-line regimen costs are identical. Second-line ART regimens, when available, cost US$669 per person-year [26]; CD4 count tests cost US$25 each [27],[28]. Tenofovir, second-line ART, and CD4 monitoring costs are each varied in sensitivity analyses.

## Results

### Prioritization by Survival Benefits (Undiscounted)

An untreated HIV-infected South African cohort starting with a mean CD4 count of 375 cells/µl (SD, 25 cells/µl) has a mean undiscounted life expectancy of 47.9 mo. A single-line stavudine-based ART regimen, initiated on development of WHO stage III/IV disease (“reference strategy”; stavudine/WHO/one-line) increases life expectancy to 99.0 mo. Table 2 provides the projected 5-y survival and life expectancies of alternative stepwise progressions toward the 2010 WHO recommendations. Compared to stavudine/WHO/one-line (step 1), 5-y survival is largest (87% survival) with the addition of CD4 count availability and ART initiation at CD4<350 cells/µl (stavudine/<350/µl/one-line). In this initial step, tenofovir/WHO/one-line (66%), stavudine/<200/µl/one-line (80%), or stavudine/WHO/two-lines (66%) each yield lower projected short-term survival. Considering each of the guideline components, stavudine/<350/µl/one-line also produces the greatest anticipated life expectancy increase, Δ25.3 mo. With stavudine/<350/µl/one-line (step 2), adding a second-line regimen results in the next largest life expectancy increase (stavudine/<350/µl/two-lines, Δ53.3 mo). The final step replaces stavudine with tenofovir (tenofovir/<350/µl/two-lines, Δ16.0 mo, step 3), resulting in a comprehensive strategy concordant with the 2010 WHO guidelines, a 5-y survival of 91%, and a projected life expectancy of 193.6 mo (Table 2).

**Table 2:** Projected life expectancies associated with alternative choices in the stepwise progression toward full implementation of the 2010 WHO HIV treatment guidelines.

Step	5-y Survival (%)	Projected Life Expectancy (Months)	Δ Projected Life Expectancy (months)^a^
**Step 1: begin with stavudine/WHO/one-line (reference strategy) (four options)**	65	99.0	—
(1) Switch from stavudine to tenofovir, or	66	112.9	13.9
(2) Add CD4 monitoring capacity, initiate ART at CD4<200 cells/µl, or	80	115.6	16.6
(3) Add second-line ART regimen, or	66	121.4	22.4
**(4) Add CD4 monitoring capacity, initiate ART at CD4<350 cells/µl**	**87**	**124.3**	**25.3**
**Step 2: begin with stavudine/<350/µl/one-line (two options)**	87	124.3	—
(1) Switch from stavudine to tenofovir, or	89	144.8	20.5
**(2) Add second-line ART regimen**	**91**	**177.6**	**53.3**
**Step 3: begin with stavudine/<350/µl/two-lines (one remaining option)**	91	177.6	—
** (1) Switch from stavudine to tenofovir**	**91**	**193.6**	**16.0**

We use the following nomenclature to define the strategies: nucleoside analog used in first line/ART initiation criterion/number of available regimens. All strategies with initiation criteria indicated by a CD4 count threshold assume availability of CD4 count monitoring. For each step, the option that maximizes survival is shown in bold.

^**a**^Change relative to the program selected in the previous step.

**Figure 2:**
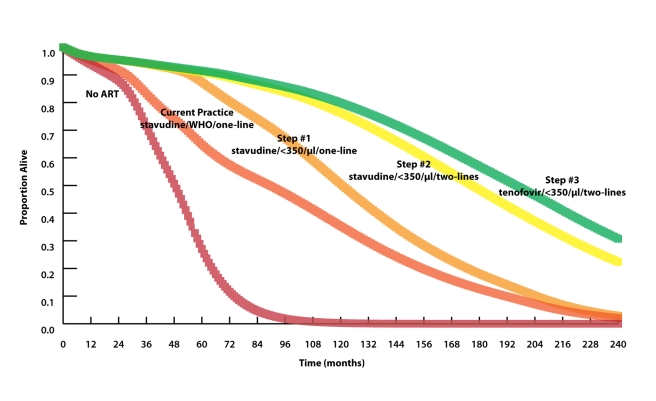
Model-projected survival curves. Model-projected survival curves (undiscounted) of the reference strategy (stavudine/WHO/one-line) and the three strategies projected to maximize life expectancy in stepwise progression toward the 2010 WHO guidelines (see Results and Table 2 for details). Curves highlighting outcomes over the next 5 y are provided in Figure S4. The 20-y horizon is presented here, not to imply that HIV treatment will remain unchanged over this time horizon, but rather to demonstrate when different interventions will have meaningful survival impacts. Median survival increases from 90 mo with stavudine/WHO/one-line (reference strategy) to 121 mo with the addition of CD4 monitoring and ART initiation at CD4<350 cells/µl (stavudine/<350/µl/one-line, step 1) to 177 mo with the addition of a second-line ART regimen (stavudine/<350/µl/two-lines, step 2). A subsequent switch from stavudine to tenofovir results in a comparatively modest survival advantage, with a median survival increase to 196 mo (tenofovir/<350/µl/two-lines, step 3). The survival curve of step 3 represents what might be expected when allthe 2010 WHO treatment guidelines are fully implemented.

Model-generated survival curves are provided for no ART, the reference strategy, and the three steps in Table 2, which act stepwise to maximize life expectancy (Figure 2). Marked differences in early survival are attributable to earlier ART initiation thresholds; differences in survival later in the disease course are associated with second-line ART availability.

### Prioritization by Cost-Effectiveness

**Figure 3:**
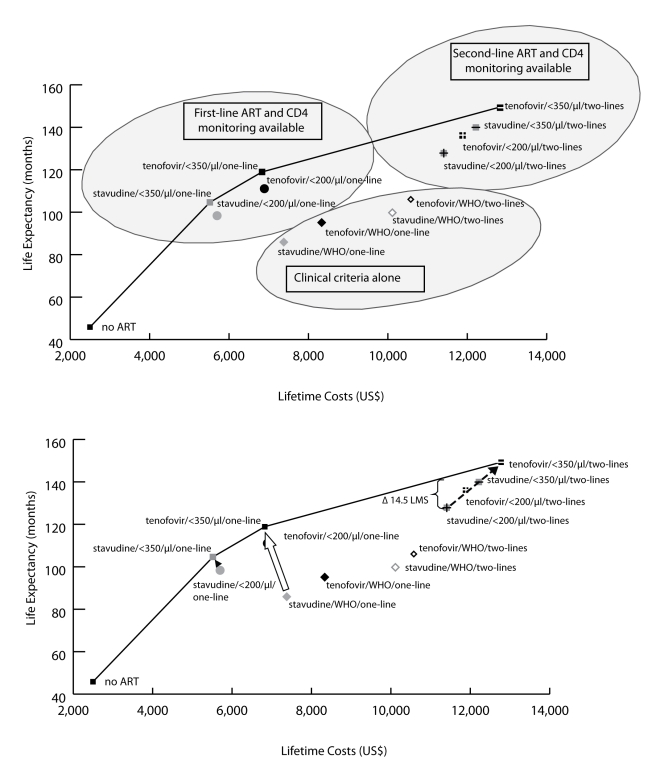
Clinical and economic outcomes of each of the scale-up interventions. The clinical and economic outcomes of all combinations of scale-up interventions are examined. The efficient frontier (marked by the line) connects the non-dominated strategies in the cost-effectiveness plane. Strategies below and to the right of the efficient frontier are those that are either strongly or weakly dominated by other options (see Methods). As illustrated in the upper panel, strategies based on clinical criteria (WHO stage III/IV) alone fall far below the efficient frontier (lower right oval), indicating their relatively high cost for the comparative benefit gained. Strategies in the upper left oval are those representing CD4 monitoring and one line of ART. Strategies incorporating a second-line regimen (upper right oval) all confer large survival benefits but at increased costs. The lower panel examines potential country situations. For instance, a country with a current stavudine/WHO/one-line policy could switch to a tenofovir/<350/µl/one-line policy (open arrow) and both decrease projected per-person lifetime costs and improve survival. A country with a stavudine/<200/µl/one-line policy could decrease per-person costs and also improve outcomes by changing to a stavudine/<350/µl/one-line policy (solid arrowhead). Countries with a stavudine/<200/µl/two-lines policy would require increased per-person expenditures to achieve the survival benefits associated with tenofovir/<350/µl/two-lines (dotted arrow). To illustrate the impact of a policy requiring that all persons receive the same intervention, we examine the arbitrary affordability threshold of US$11,500 per person. The bracket (upper right) denotes the per person survival loss (14.5 mo) attributable to a policy requiring that all persons receive the same intervention.

Incremental cost-effectiveness analysis (Table 3) reveals three non-dominated strategies (i.e., strategies that attain a given survival level by the least costly means): (1) stavudine/<350/µl/one-line (US$610/YLS), (2) tenofovir/<350/µl/one-line (US$1,140/YLS), and (3) tenofovir/<350/µl/two-lines (US$2,370/YLS). All other strategies are “dominated”—i.e., they are more expensive and confer less survival benefit than some other combination of strategies. Figure 3 (upper panel) maps the 13 strategies on a discounted cost and life expectancy plane. The line connecting the non-dominated strategies designates the “efficient frontier,” which represents both the least expensive way to attain a given survival and the maximum achievable survival for any given cost [12].

**Table 3:** Life expectancy, costs, and incremental cost-effectiveness ratios of the 12 possible stepwise combinations (and no ART) from the reference strategy to full implementation of 2010 WHO HIV treatment guidelines.

Strategy^a^	Discounted Cost	Discounted Per-Person Life Expectancy (Undiscounted) Months	Incremental Cost-Effectiveness Ratio (US$/YLS)
No ART	2,540	44.9 (47.9)	
**Stavudine/<350/µl/one-line (step 1)**	**5,550**	**104.3 (124.3)**	**610**
Stavudine/<200/µl/one-line	5,740	97.3 (115.6)	Dominated^b^
Tenofovir/<350/µl/one-line	6,870	118.3 (144.8)	1,140
Tenofovir/<200/µl/one-line	6,930	109.9 (133.9)	Dominated^b^
**Stavudine/WHO/one-line (reference strategy)**	**7,440**	**84.5 (99.0)**	**Dominated** b
Tenofovir/WHO/one-line	8,400	93.9 (112.9)	Dominated^b^
Stavudine/WHO/two-lines	10,140	98.8 (121.4)	Dominated^b^
Tenofovir/WHO/two-lines	10,640	105.0 (131.2)	Dominated^b^
Stavudine/<200/µl/two-lines	11,460	127.0 (161.3)	Dominated^c^
Tenofovir/<200/µl/two-lines	11,930	135.3 (175.5)	Dominated^c^
**Stavudine**/<**350/µl/two-lines (step 2)**	**12,270**	**138.7 (177.6)**	**Dominated** c
**Tenofovir/<350/µl/two-lines (step 3)**	**12,820**	**148.4 (193.6)**	**2,370**

The reference strategy and the strategies selected in the stepwise progression in Table 2 are shown in bold.

a We use the following nomenclature to define the strategies: nucleoside analog used in first line/ART initiation criterion/number of available regimens. All strategies with initiation criteria indicated by a CD4 count threshold assume availability of CD4 count monitoring; WHO indicates WHO stage III/IV disease.

b Strongly dominated (more expensive but confer less clinical benefit than some other strategy) [12].

c Weakly dominated (more expensive but confer less clinical benefit than some combination of other strategies) [12].

Thus, a country with a current stavudine/WHO/one-line policy (Figure 3, lower panel) could switch to a tenofovir/<350/µl/one-line policy (open arrow) and thereby simultaneously decrease projected per-person lifetime costs and improve survival. Similarly, a country with a stavudine/<200/µl/one-line policy could decrease per-person costs and also improve outcomes by changing to a stavudine/<350/µl/one-line policy (solid arrowhead). Countries with a stavudine/<200/µl/two-lines policy would require increased per-person expenditures to achieve the survival benefits associated with tenofovir/<350/µl/two-lines, as suggested in the revised WHO guidelines (dotted arrow).

### Evaluating the Cost of Equity

Of the three efficient programs (Table 3; Figure 3), tenofovir/<350/µl/one-line has a projected per-person lifetime cost of US$6,870, and tenofovir/<350/µl/two-lines has a projected lifetime cost of US$12,820. An HIV program budget that allows for a per-person cost between US$6,870 and US$12,820 might be achieved in several ways; two are illustrative. The first would be to proportionately divide the cohort between two of the programs along the efficient frontier, so that part of the cohort receives tenofovir/<350/µl/one-line and the rest receives tenofovir/<350/µl/two-lines. An alternative would be to provide everyone in the cohort a third program—one that lies below the efficient frontier. The opportunity cost (e.g., the anticipated net loss in discounted life expectancy associated with an alternative strategy choice) of any non-efficient strategy may be quantified by measuring its vertical distance from the efficient frontier. To illustrate this opportunity cost, we take an arbitrary affordability threshold of US$11,500 per person. In the example of a program that can afford no more than US$11,500 per person (stavudine/<200/µl/two-lines; Figure 3, lower panel), the opportunity cost of uniformity in care (“equity”) is 14.5 mo per person of survival (shown by the bracket in Figure 3, lower panel).

### Sensitivity Analyses

#### Clinical parameters

In sensitivity analyses, we examine changes in clinical input data required to alter the stepwise ordering of program additions. Modest reductions in the mean CD4 count of the cohort (to 250 cells/µl) show decreased clinical benefits to earlier ART initiation but no substantial changes in cost-effectiveness. When the mean CD4 count of the cohort is less than 100 cells/µl, the benefits of a policy change to earlier ART initiation are largely irrelevant (Text S2). This is because the majority of the cohort is already ART-eligible regardless of the initiation criterion (WHO stage III/IV disease, CD4<200 cells/µl, or CD4<350 cells/µl). Although CD4 monitoring still improves cohort survival compared to clinically based ART initiation, in populations with mean CD4 counts far below the policy-relevant ART initiation criteria, the addition of a second-line regimen becomes the most clinically beneficial intervention. For the anticipated life expectancy benefits of tenofovir/WHO/one-line to exceed those expected with stavudine/<350/µl/one-line, replacement of stavudine with tenofovir would have to increase the 24-wk suppressive efficacy from 85% to 95% and simultaneously decrease the monthly probability of later virologic failure by 50% (from 0.01 to 0.005) (Text S3) [24]. Second-line ART maintains its position in the stepwise order (step 2) as long as its 24-wk viral suppression rate remains between 40% and 88%, even with a 3-fold increase in the rate of late failure when efficacy decreased to 40% (Text S3). Increasing stavudine toxicity by 2-fold alters life expectancy estimates by less than 1 mo and does not change the recommended stepwise additions (Text S3). Similarly, changes in the gender distribution of the cohort have little impact on the results (Text S3).

#### Cost parameters

Holding efficacy constant, results are very sensitive to the price of tenofovir; a decrease in the cost of tenofovir from US$135 to US$51 per person per year would make tenofovir both more effective and less costly than stavudine. Results are less sensitive to the costs of second-line regimens (15% of base case) and CD4 monitoring (three times base case), neither of which produced meaningful changes in cost-effectiveness results (Text S2). In two-way sensitivity analyses, where the cost of tenofovir is decreased and its efficacy increased, tenofovir/<350/µl/one-line dominates stavudine/<350/µl/one-line when the tenofovir regimen costs are US$153 annually (75% of the base case) and its 24-wk suppressive efficacy is 90% (5% increase from the base case).

#### Additional sensitivity analyses

Further sensitivity analyses are detailed in the Texts S2 and S3. In Text S2, we present the 1- through 5-y survival rates for all 12 strategies examined, as well as the survival curves of the stepwise strategies selected on a 5-y, rather than a 10-y, horizon (Figure S4). Text S2 also provides the details of analyses under conditions of alternative mean CD4 counts for the cohort and alternative costs of both second-line regimens and CD4 monitoring. Further analyses (Text S3) offer additional comprehensive analytic variations in cohort gender distributions, ART initiation criteria, first- and second-line ART efficacies, stavudine-related toxicities, and costs. Within plausible ranges, these sensitivity analyses, other than those reported above, had little impact on clinical- or policy-relevant results.

## Discussion

The new 2010 WHO ART guidelines aim to promote public health interventions that “secure the greatest likelihood of survival and quality of life for the greatest number” of individuals with HIV. The reported guiding principles in the revision process include: (1) do no harm, (2) ensure access and equity, (3) promote quality and efficiency, and (4) ensure sustainability. Motivated by these tenets, the new guidelines recommend a single CD4-based ART initiation criterion for all populations, a switch from stavudine to tenofovir, and universally available second-line regimens [2]. We find that in settings where immediate implementation of all of the new WHO treatment guidelines is currently not feasible, ART initiation at CD4<350 cells/µl provides the greatest short- and long-term survival advantage and is very cost-effective. In countries that are already initiating stavudine at CD4<350 cells/µl and have access to CD4 monitoring, switching from stavudine to tenofovir increases survival and is also cost-effective. Access to second-line ART provides more clinical benefit than access to tenofovir but at substantially greater costs.

The additional outlays implied by the new guidelines stand in stark contrast to the resource-constrained reality encountered on the ground. Many countries are still striving to meet goals set by the now-superseded 2006 guidelines. The WHO estimates the current ART coverage rate across low- and middle-income countries to be 42% [5],[29]. Meanwhile, the new guidelines recommend access to CD4 count monitoring, call for treatment of almost double the 3–5 million people already requiring treatment based on the previous guidelines [30], and suggest replacement of the most widely used antiretroviral drug with one that costs nearly US$100 per patient-year more [26]. In most resource-limited settings, the relevant policy questions are: *What is feasible now?* and *What to do first?*

Based on projected short- and long-term survival and cost-effectiveness results, we identify three critical messages. First, countries with very limited resources and still only one line of ART available should focus first on access to CD4 count monitoring and ART initiation at CD4<350 cells/µl. These should be implemented before switching from stavudine to tenofovir and prior to providing second-line ART. Although advising to use stavudine in the first-line regimen—with its inherent toxicities—may be seen as conflicting with the primary WHO principle “first, do no harm,” the switch from stavudine to tenofovir is the recommendation that provides the least overall increase in survival, according to the results presented here. Initiating stavudine-based ART at CD4<350 cells/µl, compared with clinically based ART initiation, provides immediate and substantial short-term survival benefits, yields the greatest life expectancy compared to other guideline components, and is cost-effective by international standards. In cases where most patients present to care with CD4 counts far below the ART initiation threshold (e.g., CD4<100 cells/µl), a policy of earlier ART initiation is neutral at worst—both in terms of cost and clinical outcomes—as it serves only to increase life expectancy among patients with less advanced disease.

Second, countries with currently one line of ART available but more resources should ensure access to CD4 count monitoring with ART initiation at CD4<350 cells/µl and then switch from stavudine to tenofovir, before making second-line ART available. Indeed, some countries have already responded to the 2010 WHO guidelines and have made plans to phase out stavudine [31]. Reductions in the price of tenofovir could resolve the ongoing debate surrounding the role for stavudine in resource-limited settings. At an annual cost of US$51, tenofovir would be both less costly and more effective than stavudine.

Third, in countries with sufficient budgets to provide second-line ART, it is neither effective nor cost-effective to maintain stavudine in first-line regimens. Second-line ART may offer additional efficiencies by decreasing the prevalence of resistant virus and leaving future drug regimen options available.

Once countries have the capacity to provide early ART initiation, tenofovir, and second-line regimens, there will be additional clinical and policy questions. Policy makers will be addressing what to do upon second-line failure; optimal third-line regimens will be in question. Expanded ART regimen availability leads to clinical need for timely ART switches and forces the issue of HIV RNA laboratory availability. Finally, timely ART initiation is currently limited by late presentation to care [32],[33]. Concurrent with scaling up to achieve the 2010 WHO ART guidelines, there should be a concerted effort to achieve the 2007 WHO HIV screening guidelines [34]; without earlier case detection, a policy of ART initiation at CD4<350 cells/µl will never be effectively realized.

It is important to highlight that full and immediate implementation of the comprehensive set of new guidelines is cost-effective by South African standards. But, while it is helpful to critically examine the survival and economic efficiency of alternative programmatic choices, “cost-effective” does not mean “affordable.” In the setting of clear budget constraints, the question of affordability may conflict with the political imperative that all persons receive the same care package. In this case, prioritization of equity over efficiency decreases mean life expectancy—sometimes by more than 1 y per person—for the same healthcare expenditure (Figure 3, lower panel).

This analysis has several limitations. We report results from a cohort of HIV-infected individuals initiating ART. Although we believe the overall results would be consistent, this analysis does not specifically address ART programs with patients already in alternative stages of care, including some on first-line regimens, some on second-line regimens, and some who have previously accumulated drug-related toxicities. Such diversity within a cohort would require more individualized analyses. Additionally, a full budget impact analysis would be required to examine the number of patients in need of care, and to project the implications of each component of the WHO recommendation on program budgets over alternative time horizons.

Despite its limitations, this analysis represents the only systematic, scientific effort we are aware of that marshals the evidence base in support of implementing the WHO guidelines. The most unfortunate outcome upon release of the revised WHO guidelines would be either their complete dismissal on cost grounds alone, or the execution of more expensive—though easier to implement—interventions that offer less overall health benefit than other interventions.

In cases where the simultaneous implementation of all components of the 2010 WHO ART guidelines is beyond the reach of programs or countries, important prioritization questions emerge. This analysis suggests that CD4 count monitoring and ART initiation at CD4<350 cells/µl are the critical initial priorities. Replacing stavudine with tenofovir would further increase survival and would also be cost-effective. Adding a second-line ART regimen would provide large survival benefits, but with substantial increases in the necessary budgets.

## Supporting Information

Figure S1 ART scale-up strategies.
(0.62 MB DOC)

Figure S2 Course of disease.
(0.22 MB TIF)

Figure S3 Validation of South African natural history data in the CEPAC model.
(0.33 MB TIF)

Figure S4 Patient survival in the first 5 y after model entry.
(0.41 MB TIF)

Text S1 WHO priorities: Technical appendix.
(0.27 MB DOC)

Text S2 WHO priorities; sensitivity analyses addendum, part 1.
(0.26 MB DOC)

Text S3 WHO priorities; sensitivity analyses addendum, part 2.
(0.12 MB XLS)

## Abbreviations

ART: antiretroviral therapy
CEPAC: Cost Effectiveness of AIDS Complications
SD: standard deviation
WHO: World Health Organization
YLS: years of life saved
