# Supplementary figures and images for: Scaling Up the 2010 World Health Organization HIV Treatment Guidelines in Resource-Limited Settings: A Model-Based Analysis
Source: PLoS Med. 2010 Dec 21;7(12):e1000382. doi: 10.1371/journal.pmed.1000382 (PMC3014084; doi:10.1371/journal.pmed.1000382)

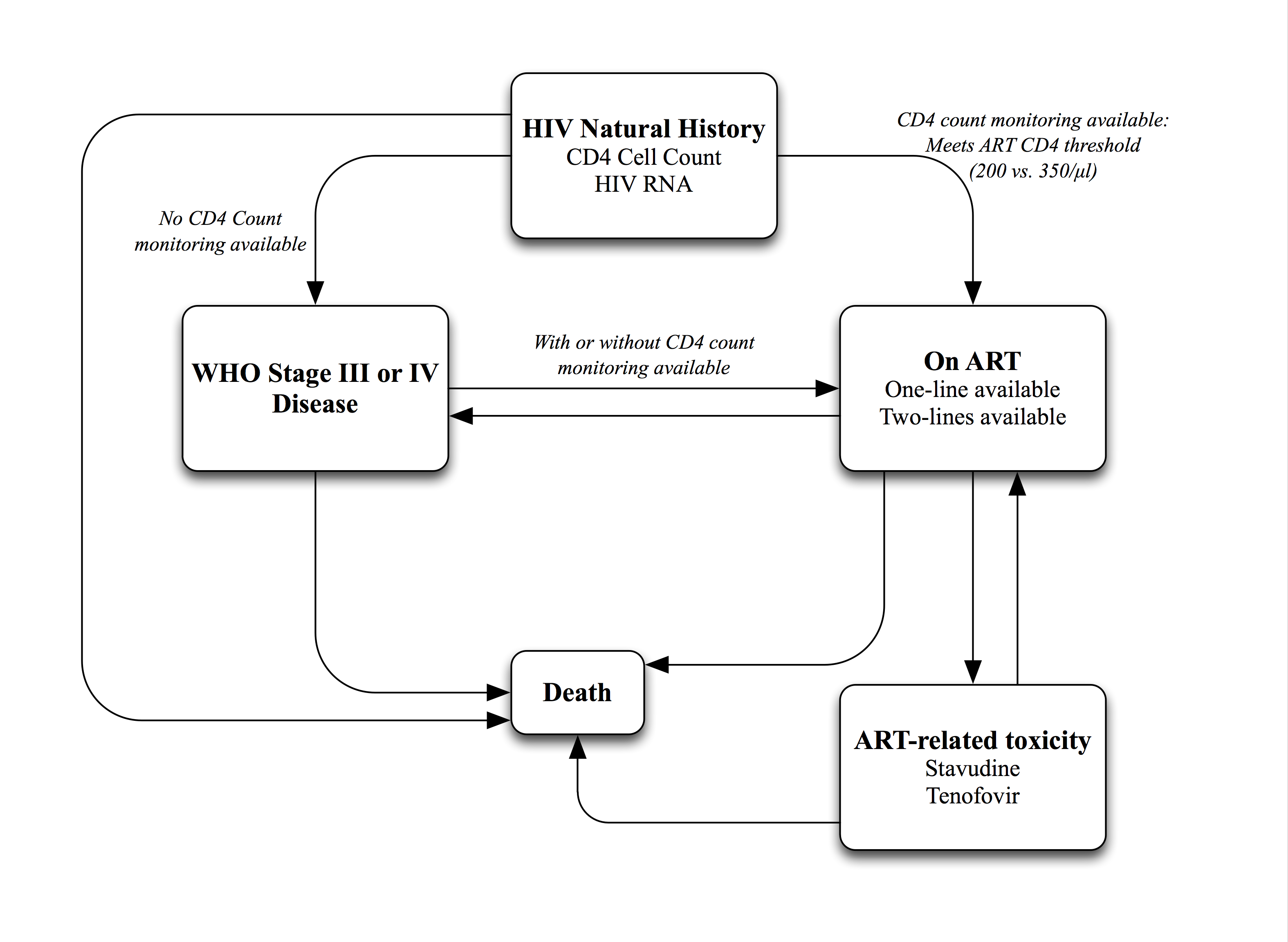

Supplement: Figure S1 — ART scale-up strategies. (0.62 MB DOC) [file pmed.1000382.s001.tif]

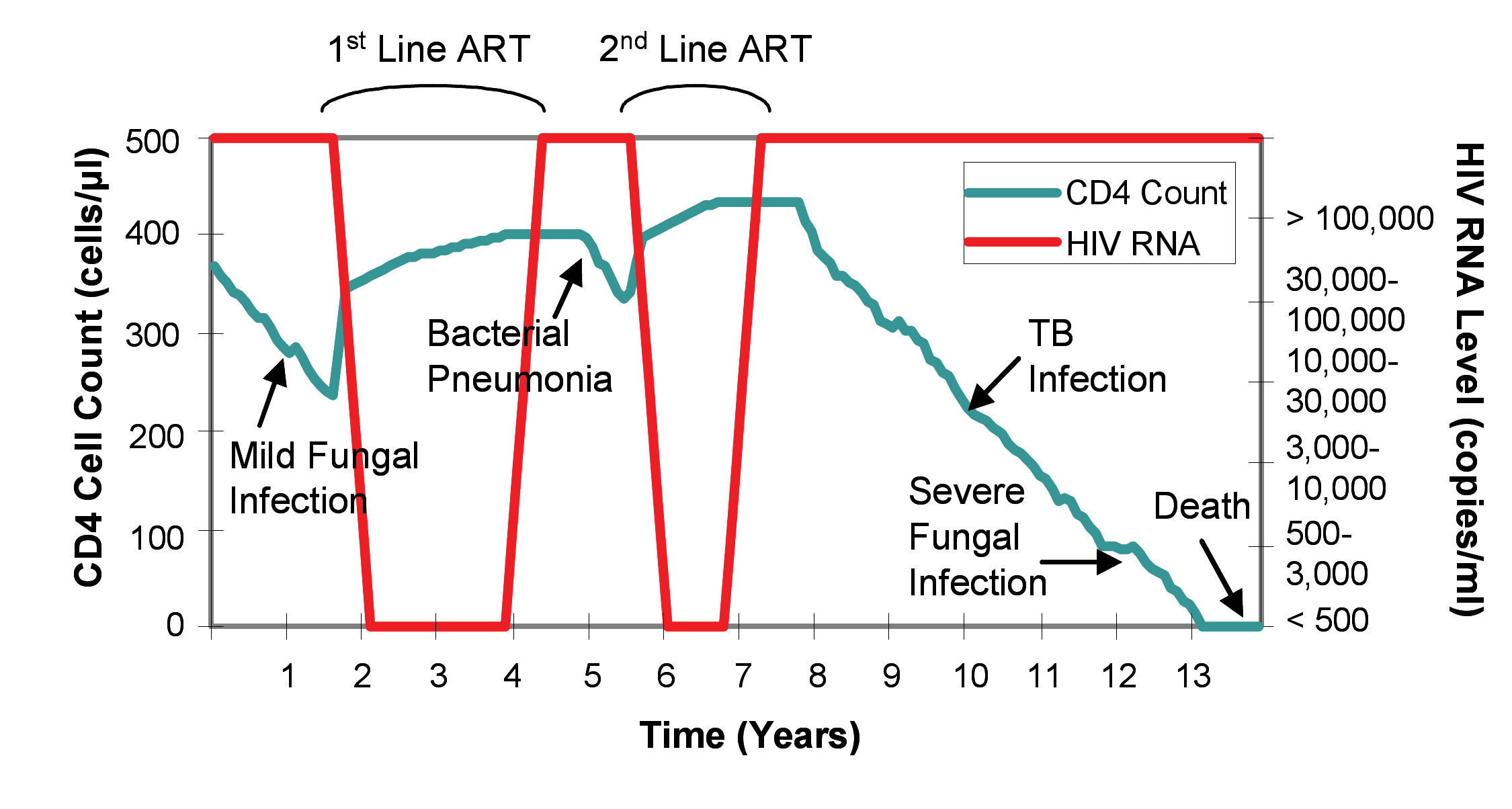

Supplement: Figure S2 — Course of disease. (0.22 MB TIF) [file pmed.1000382.s002.tif]

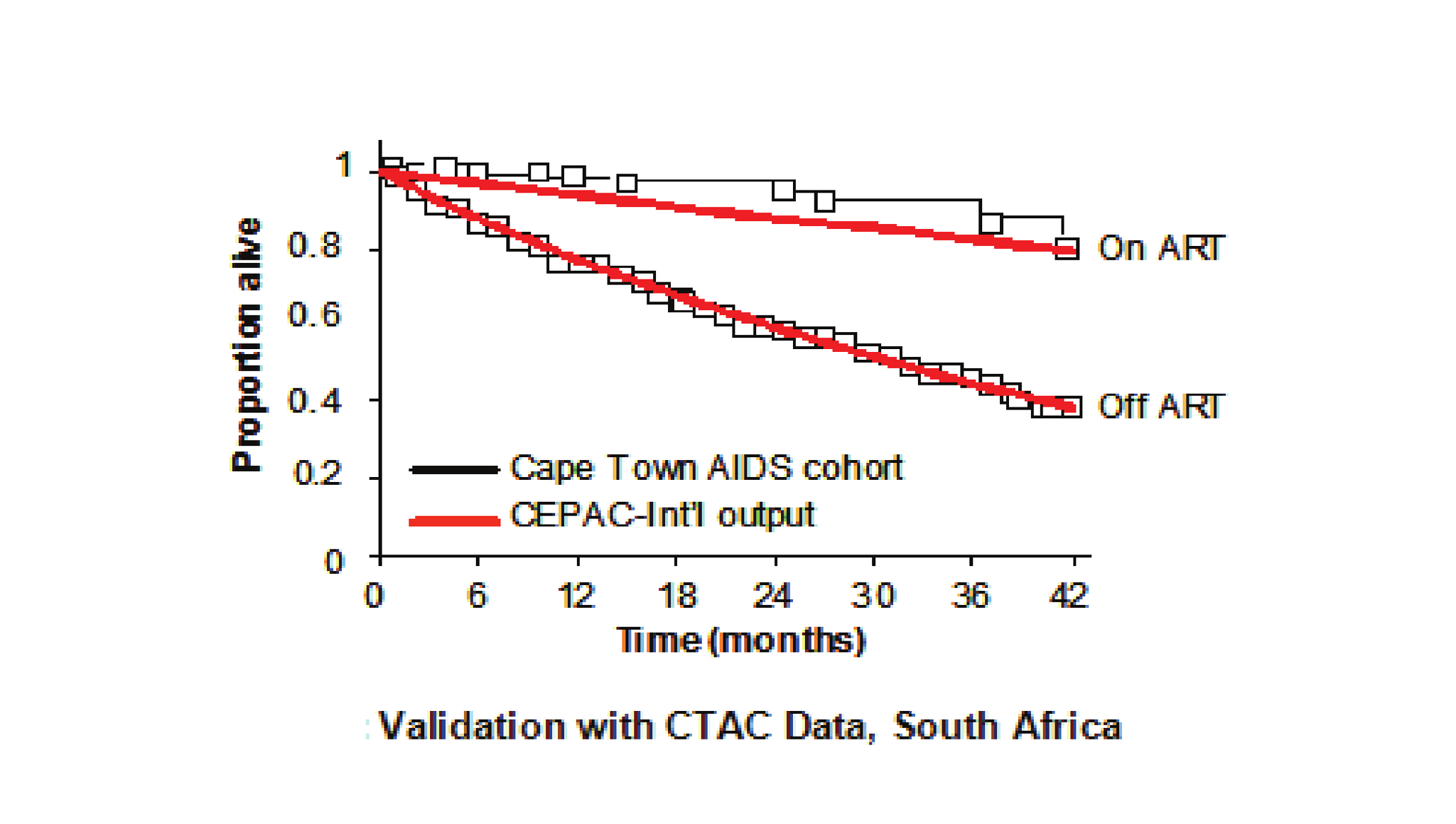

Supplement: Figure S3 — Validation of South African natural history data in the CEPAC model. (0.33 MB TIF) [file pmed.1000382.s003.tif]

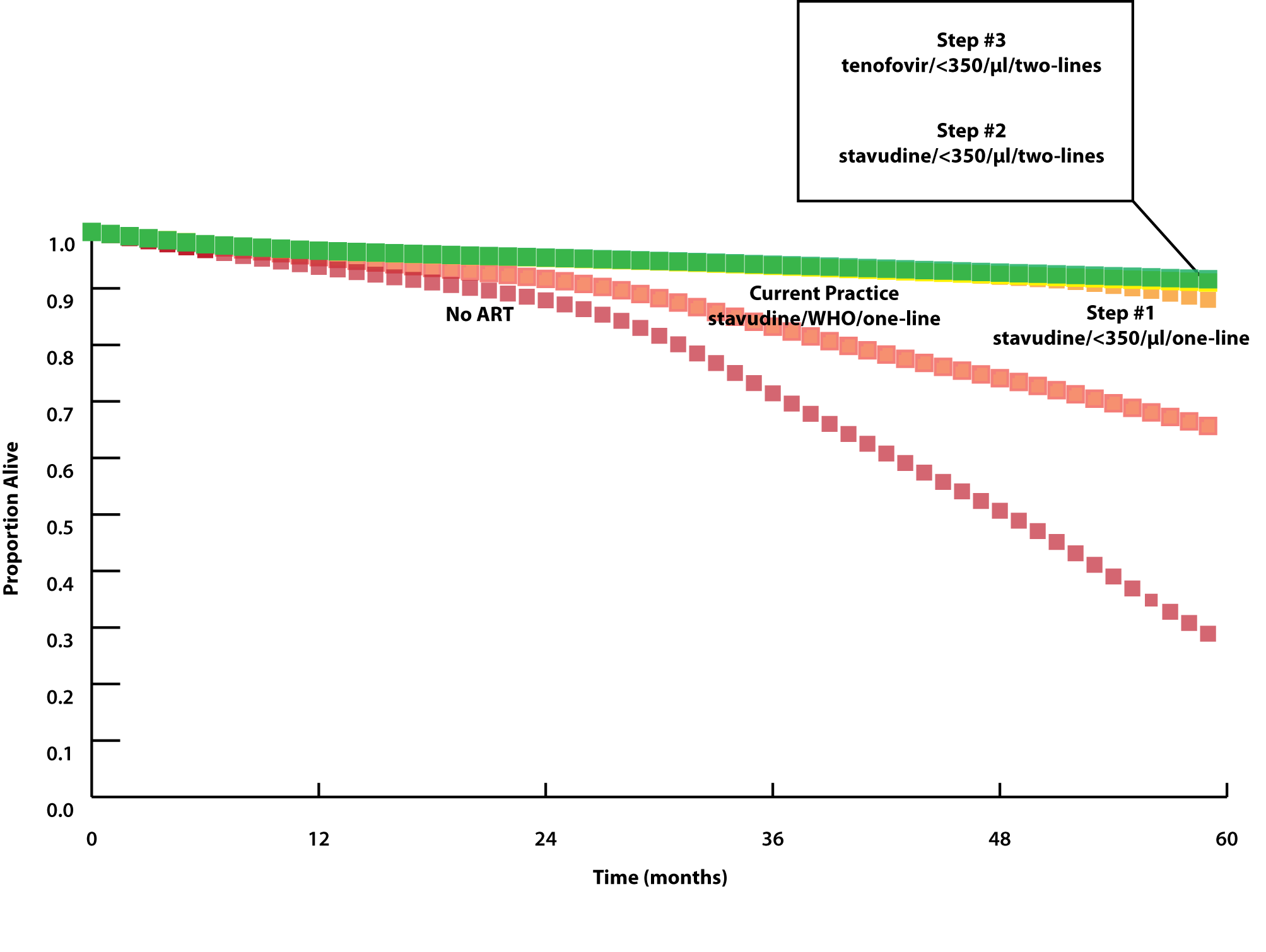

Supplement: Figure S4 — Patient survival in the first 5 y after model entry. (0.41 MB TIF) [file pmed.1000382.s004.tif]
