# Supplementary material for: Scaling Up the 2010 World Health Organization HIV Treatment Guidelines in Resource-Limited Settings: A Model-Based Analysis
Source: PLoS Med. 2010 Dec 21;7(12):e1000382. doi: 10.1371/journal.pmed.1000382 (PMC3014084; doi:10.1371/journal.pmed.1000382)
Supplement: Text S1 — WHO priorities: Technical appendix. (0.27 MB DOC) [file pmed.1000382.s005.doc]

**Scaling up the 2010 WHO HIV treatment guidelines in resource-limited settings:**

**A model-based analysis**

**Technical Appendix**

**Walensky et al.**

**This appendix provides technical detail on the modeling framework and methods that have supported a variety of previously published analyses. It is divided into 2 sections: 1) a description of model methods; and 2) detailed input parameters (largely in Table TA 2) to complement Table 1 of the main paper. We also direct interested readers to the CEPAC website, which offers further model details, including flowcharts, mathematical formulas, and our processes for maintaining updated input parameters from the literature:** [**http://web2.research.partners.org/cepac/**](http://web2.research.partners.org/cepac/)**.**

**INTRODUCTION**

The purpose of this technical appendix is to offer further detail on the methods, input data, and results described in the main body of the manuscript, Walensky et al. *Scaling up WHO recommendations for HIV therapy in resource-limited settings: What to do first.*  The appendix has three parts: the Methods section provides additional detail on cost-effectiveness analysis and the CEPAC International model; the Data section describes the sources and derivation of input parameters used for the analysis; the Results section offers additional figures and a more exhaustive review of the sensitivity analyses we conducted to establish the robustness of our conclusions.

**METHODS**

**Reported Outcomes and Cost-effectiveness**

The model estimates mean per person life expectancy and mean per person lifetime cost. The incremental cost-effectiveness ratio was calculated for each strategy; strategies that were “strongly dominated” (more costly but less effective than an alternative strategy) and those that were “weakly dominated” (had a higher cost-effectiveness ratio than the next more effective strategy), were excluded and cost-effectiveness ratios were recalculated [1, 2]. Incremental cost-effectiveness ratios are generally expressed as $/quality-adjusted (or disability-adjusted) years of life saved. Analyses in resource-limited settings are often reported without quality-adjustment; this is justified by the paucity of utility data in these settings. We adopted a modified societal perspective, excluding patient time and travel costs, and discounted both costs and life expectancy at 3% per year to report incremental cost-effectiveness ratios [1]. The Data section, cost subsection, provides further details; the model includes HIV-associated direct medical resource utilization (including outpatient visits, inpatient days, laboratory tests and medication costs) but excludes direct non-medical costs and indirect costs (i.e. patient time and lost wages).

In 2001, the WHO-sponsored Commission on Macroeconomics and Health proposed that “cost-effectiveness” be defined in relation to an individual country’s annual *per capita* gross domestic product (GDP) [3]. When Health interventions with incremental cost-effectiveness ratios less than three times the annual *per capita* GDP in a country were considered “cost-effective” for that country, while interventions with cost-effectiveness ratios less than the annual *per capita* GDP itself were considered “very cost-effective.” As a reference point for this analysis, we compared the results to the 2008 *per capita* GDP in South Africa, which was US$5,700 [4].

In addition to life expectancy and total lifetime costs, model outputs may include disaggregated estimates of average cost per patient (e.g., drug, laboratory, hospitalization), performance of therapy (e.g., time on therapy, time to virologic rebound) and morbidity (specific OD incidence rates, and causes of death).

**Analytic Overview**

The objective of this analysis is to examine, and help prioritize, every feasible sequence of scale up strategies toward the 2009 WHO ART guidelines. We begin with an assumed baseline level of care (designated the “reference strategy”) which we take as a one-line stavudine-containing ART regimen, initiated at WHO Stage III or IV disease, without CD4 count monitoring capacity. From this reference strategy, we examine all possible combinations of the following scale up components: 1) widespread CD4 count monitoring capacity, allowing for ART initiation at CD4 <200/µl (and biannual monitoring); 2) earlier ART initiation, at CD4 <350/µl (presumes CD4 count availability); 3) an available second-line ART regimen upon first-line failure; and 4) replacement of stavudine with tenofovir in the first-line regimen. To reference these strategies, we use the following nomenclature: nucleoside analogue in first-line/ART initiation criteria/number of regimens [e.g. stavudine/<200/µl/two-lines]. The combined implementation elements result in twelve possible strategies, in addition to no ART. These strategies are outlined in the paper (Figure 1) and are enumerated in the Table TA 1. Figure S1 is a bubble diagram of the ART scale up strategies as applied to the CEPAC International Model. As the model specifications below will describe, not only must patients meet criteria for ART initiation – however those criteria are designated – but they must also be “observed” to have met those criteria. For example, a CD4 decline to <200/µl will under trigger the initiation of ART under such policy, when a CD4 cell count test is obtained and the results received.

**CEPAC International**

The CEPAC International Model is a computer-based, state-transition, Monte Carlo simulation model of the progression and outcomes of HIV disease in a hypothetical cohort of patients in resource-limited settings. “State-transition” means that the model characterizes the natural history of illness in an individual patient as a sequence of monthly transitions from one “health state” to another. “Monte Carlo” refers to a random number generator and set of estimated probabilities that are used to determine the sequence of movements between health states for a particular patient. Each individual patient’s clinical course is followed from the time of entry into the model until death. A running tally is maintained of all clinical events, the length of time spent in each health state, and the costs associated with each health state. Upon the patient’s death, summary statistics are recorded and a new patient enters the model. This process is then repeated for a large number of simulated patients (statistical convergence can typically be achieved with cohort sizes of one million), at which point overall performance measures such as average life expectancy and cost are computed.

In the Disease Model, health states are chosen to be descriptive of the patient’s current health, relevant history, and resource utilization patterns. They are designed to be predictive of clinical prognosis, including disease progression, immune system deterioration, development and relapse of different opportunistic diseases (ODs), toxic reactions to medications, resistance to therapy, and mortality. The model defines three general categories of health states: chronic, acute, and death. Most of the time, patients are in one of the chronic states, where progression of disease and immune system deterioration (CD4 decline) take place. Patients who develop an acute complication (e.g., an OD or drug-related toxicity) temporarily move to an acute health state, where both resource consumption levels and mortality rates are higher. Deaths can occur from either a chronic or an acute state and can be attributed to a particular OD, chronic AIDS (e.g., wasting), or non-AIDS-related causes.

The chronic and acute health states are stratified by: actual current and nadir CD4 cell count (>500 cells/µl; 301–500 cells/µl; 201–300 cells/µl; 101–200 cells/µl; 51–100 cells/µl; and 0-50 cells/µl) and current and set-point HIV RNA level (>30,000 copies/mL; 10,001–30,000 copies/ mL; 3,001–10,000 copies/ mL; 501–3,000 copies/ mL; 51-500 copies/ mL; 0-50 copies/mL). Drawing from distributions of patient characteristics (age, sex, CD4 count and HIV RNA level) derived from South Africa, a patient is randomly assigned to a health state **upon model entry**. By permitting the user to define initial population distributions for patient age, sex, CD4 cell count, HIV-RNA, and other demographic and clinical attributes, the model has the flexibility to explore a broad range of different patient cohorts.

At the start of each one-month cycle, the model records the patient’s CD4 count, HIV-RNA level, history of acute illness, and current therapies and uses these characteristics to determine the probabilities that indicate movement to a new state in the subsequent month. Monthly probabilities of events are estimated directly from published sources and available databases and translated into risk functions for the model [5, 6]. These risk functions embody the key parameters of the natural history of HIV illness, AIDS, and ODs, including: rates of disease progression, OD risks, survival probabilities, and the effects of therapy. The model treats HIV RNA as the primary driver of immune system deterioration, and thus the assigned viral load level determines the rate at which the patient’s CD4 count will decline in the absence of ART [7]. Recognizing the absence of data suggesting that HIV RNA levels differ by geographic region or clade, we have validated that these US-based data provide a reasonable surrogate for the dynamics between CD4 count and HIV RNA in the African setting [8]. Patients with a history of ODs have a higher risk of recurrence, depending on CD4 count and current use of antiretroviral therapy [5].

We are careful to distinguish in the model “actual” CD4 cell count and HIV RNA – i.e. the underlying immunologic and virologic state, regardless of whether it is measured by a laboratory test– from “observed” CD4 cell count and HIV RNA – that which is measured by a test and upon which clinical decisions can be made. Clinical events within the model are predicated on patient’s “actual” CD4 count and viral load status. In resource-limited settings, however, this information may not be available to the provider due to limited access to laboratory monitoring. The International Model has the flexibility to represent decision making based on data available including: 1) clinical events alone; 2) CD4 count monitoring (with variable frequency); and/or 3) HIV RNA monitoring (with variable frequency).

*Clinical Visits and Laboratory Monitoring*

Upon entry to the model, all patients undergo a clinic visit to observe their initial OD histories. If CD4/HIV RNA monitoring is available, these tests may also occur at this time. At this initial visit, if specific criteria are met, patients will initiate prophylaxis and antiretroviral therapies. Subsequent clinic visits will then be scheduled at regularly specified intervals.

In addition to the regularly scheduled clinic visits, certain events (ODs) may trigger an emergency clinic visit to occur in that month. An emergency clinic visit is associated with the same costs and clinical decision-making opportunities as a routine visit, and at most one clinic visit can occur in a given month.

If the user specifies a strategy with CD4 and/or HIV RNA monitoring available, these tests are generally administered at the time of a clinic visit. Standard testing frequency may be user-defined; other conditions may also trigger additional tests, including observed ART failures that require confirmation by CD4 or viral load, depending on the confirmation method specified by the user.

*ART and ART Efficacy*

The model has capacity to simulate up to ten lines of ART, to be administered sequentially. The current analysis, focused in South Africa, utilizes only 2 sequential regimens. A patient will be evaluated for starting, stopping, or switching the ART regimen at every clinic visit. The criteria for regimen change can be specified differently for each individual regimen. Upon meeting criteria for ART initiation (or switching), the patient will be started on the first (or next) specified regimen.

A patient may be evaluated to start an ART regimen based on the following criteria – current CD4 count (if observable data available), current HIV RNA (if observable data available), a combination of CD4 count and HIV RNA, observed ODs since the previous regimen, or CD4 count and observed ODs. Since this structure enumerates the logical combinations of the individual criteria, they can be independently specified and evaluated. If the specified criteria are met, the patient will be started on that ART regimen. The first regimen criteria are evaluated at each clinic visit until treatment is initiated.

The model’s handling of efficacy and durability of antiretroviral therapy is as follows: we estimate efficacy from data on viral suppression and CD4 count change over time, as reported in cohort studies and randomized trials [9, 10]. From these data, we derive a probability of “early” (within six months) and “late” (beyond six months) failure for each first-line and second-line antiretroviral regimen (and subsequent regimens) to be considered. This structure allows patients to transition from virologic suppression to “failure” with the appropriate change in HIV RNA and CD4 count. Virologic suppression results in CD4 increases, also in these two time phases, that occur in concordance with data reported in clinical trials [9, 10]. An initial large CD4 benefit occurs in the “early” period, followed by a modest benefit that occurs over a longer time horizon, as long as the patient remains virologically suppressed.

The model makes a distinction between patients actually failing an ART regimen and those who are observed to fail a regimen. The former can be regarded as patients in whom therapy stops providing any substantive biological benefit to the patient. The latter simulates the clinical observation of a new OD, or laboratory detection of CD4 decline or viral load increase, indicating a regimen’s lack of continued benefit, at which point the patients may be taken off that regimen.

***Toxicity***

Patients on ART are subject to drug toxicities that fall into one of three categories: acute minor toxicity, acute major toxicity, and chronic toxicity. All three types of toxicities result in additional patient care costs. Major and chronic toxicities confer increased risks of mortality. Minor and major acute toxicities are modeled to have only a one-month duration, while chronic toxicities are simulated to persist for user-defined periods of time.

Drug toxicities are specified by their probability, their time to occurrence, their duration and their costs. ART toxicity results in the use of “subregimens” -- interchangeable combinations of drugs within an ART regimen. Subregimens model the practice of substituting individual drugs of the same class for prevention of, or in response to the occurrence of, a specific toxicity. For each of the toxicities that occur during a particular subregimen, the user can specify which subregimen the patient will switch to in response. The occurrence of a toxicity results in an emergency clinic visit during which the drug switch occurs.

***Costs***

In each month, patients accrue a monthly routine care cost, based on their current CD4 and OD history state [5, 11, 12]. If a patient has a history of an OD, the cost is based on that OD type and the patient’s CD4. In addition to these base routine monthly costs, patients accrue costs for each type of acute event (e.g. acute OD, toxicity, visits, tests, death) as well as monthly drug (i.e. ART, OD prophylaxis) costs.

**Note on Discrete Events and Monthly Cycles**

The basic unit of time in the model is a month. All acute events in the model occur for durations of time much smaller than a month. The result is that there are cases where the accounting of discrete events may not make much sense at initial inspection. (The halving of costs in a month of death is one simple manifestation of this issue.) The scheduling of tests is directly affected by the timing of discrete, acute events.

**Monthly Cycle of the Model**

Because all events in the program occur discretely, it is important to keep in mind the order of evaluation in each month of a simulated patient. Taking all the mechanisms described above together for HIV-infected patients, each regular monthly cycle in the program involve the following steps in order:

1. increase the patient’s age, in months
2. compute whether a CD4 and/or HVL test(s) should be performed this month, based on last tests
3. if a CD4 test is to be performed, do so
   1. start and stop OI prophylaxes as necessary based on the patient’s new observed CD4
4. if a viral load test is to be performed, do so
   1. if the patient is on an ART, see if the new observed viral load (and CD4) results in a failed ART diagnosis
      1. if the number of repeat failure diagnoses is enough for a confirmed failure, take the patient off the ART regimen
      2. if the patient has not actually failed the ART regimen (i.e. the failure diagnoses are incorrect), treat the patient’s CD4 and HVL levels as in ART failure to reflect the discontinuation of the effective therapy
5. if the patient is not on any ART regimen and qualifies for a new line of ART (per observed CD4 and HVL, number of lag months between ARTs, etc.), start the patient on the next regimen
6. for all prophylaxes the patient is currently on, see if an associated toxic event occurs
7. if the patient is on an ART regimen, see if an associated toxic event occurs
   1. if an ART major toxicity event occurred, see if it also caused death – if death did occur, stop the patient simulation
8. determine if the patient dies from chronic AIDS or non-AIDS causes
   1. if death occurs, stop the patient simulation
9. for each prophylaxis the patient is on, determine if resistance is to start in the current month
10. determine if an acute OI event will occur this month
    1. if an OI event occurs, see if it also causes death from the OI and thus stop the patient simulation
    2. if the patient is not detected as HIV+, make the patient detected
11. update the patient’s CD4 and HVL for the month
12. update the patient’s accumulated costs, life months, and quality adjusted life months

**DATA**

**General Approach to Data Acquisition**

We have a formal protocol for systematic review of the published literature that is conducted every few months to ensure that input parameters in the model are up to date. Online databases searched include MEDLINE, AIDSLINE, HealthSTAR, EconLit, and Cochrane, and are supplemented by manual searches of recent publications not yet indexed online, and searches of websites specific to HIV/AIDS. Because of the rapid evolution of HIV data, we also review abstracts from major international meetings (e.g. International AIDS Conference, Conference on Retroviruses and Opportunistic Infections, International HIV Drug Resistance Workshop). When multiple parameter estimates are available, we consider the strength of study design, and sample size, similarity of patient populations, and outcome measurements. If meta-analytic techniques are required to derive summary measures of parameter estimates, we conduct such analyses, as we have done for second-line ART efficacy [13]. In the event of conflicting results that cannot be resolved by meta-analytic methods, we include all appropriate studies and explore the implications of different estimates on the results of each analysis.

**Input parameters**

# This section provides further details on input parameters and is meant to complement the information provided in the main paper. Input parameters for the model are provided in abbreviated form in the manuscript Table 1. They are provided in more detail, stratified by CD4 cell count where applicable, in Technical Appendix Table TA 2.

# *Natural History*

# Incidence of ODs, death from ODs, and chronic AIDS-related deaths in South Africa were from the Cape Town AIDS Cohort (CTAC) and were stratified by CD4 count [5]. In the absence of specific data from South Africa, rates of CD4 count decline, stratified by baseline HIV RNA level, were from the Multicenter AIDS Cohort Study (MACS) in the US [7].

*Prophylactic Efficacy*

According to WHO guidelines, all patients receive co-trimoxazole prophylaxis at model entry [14]. In the absence of co-trimoxazole efficacy data from South Africa, we use efficacy data as reported in from a placebo-controlled clinical trial from Côte d’Ivoire [15]. In resource-limited settings, co-trimoxazole not only has prophylactic efficacy against *Pneumocystis jiroveci* and toxoplasmosis*,* but also against bacterial infections and other WHO stage IV defining diseases (eg. isospora). We also include the trial findings of co-trimoxazole increasing the rates of mild fungal disease.

*ART efficacy*

Stavudine-based, first-line ART efficacy is derived from a systematic literature review and meta-analysis of ART responses in Africa [9]. From this analysis, we derive model input parameters, including rates of 24-week virologic suppression (75%) and 48-week CD4 cell count response (136 cells/µl). There is a 2% monthly probability of failure after 48 weeks, which is projected using published 24-week data [9]. In the absence of reliable efficacy data of a tenofovir-based regimen in resource-limited settings, we use a virologic suppression rate of 85% at 24 weeks, as reported in clinical trials [16, 17]. Despite the improved rates of virologic suppression, we want to maintain conservative assumptions with regard to CD4 benefit among those suppressed, so we use the same benefit (136 cells/µl) as those used for the stavudine-based regimen [9]. We estimate the monthly risk of later failure as 1%/month [16, 17].

*Costs*

The analysis considered HIV-associated direct medical resource utilization, including outpatient visits, inpatient days, laboratory tests and medication costs. Direct non-medical costs and indirect costs (i.e. patient time and lost wages) were excluded. Healthcare utilization was derived from CTAC using a unit costing approach [5]. Unit costs for inpatient days ($224/day) and outpatient visits ($11/visit) were from previous reports in South Africa (Table 1) [11, 12]. Costs were analyzed according to the number of inpatient hospital days and outpatient clinic visits associated with each month of routine HIV care in the absence of ODs and with each type of OD, and for death. Estimated costs of a given service (e.g. daily inpatient costs and outpatient visits) were multiplied by the number of units of service delivered to achieve an estimated cost to be applied in the month of an OD, in the month before death, in the month of death, and for all other months (stratified by CD4 cell count) in the absence of OD or death.

**Discounting**

For analyses that report projected costs and life expectancy (to create cost-effectiveness ratios), both are discounted on a monthly basis. The program expects the discount factor to be of the form 1 + r, where r is the desired discount rate. For example, a monthly discount rate of 1% would be entered as 1.01 (i.e. 1 + 0.01) to the program. The program uses this discount factor as the divisor for all

projected costs and life months accrued by each patient. The first monthly cycle for each simulated patient is always undiscounted; subsequent months are discounted by the discount factor, compounded on a monthly basis.

The default monthly discount rate used results in an annualized discount rate of 3%; the discount factor used for the program is 1.00247, calculated by (1 + 0.03)1/12, accounting for the conversion from an annualized to a monthly basis. For debugging purposes, the discount rate is often changed to 0% – in this case, the discount factor used in the program is 1.

**Validation**

We ascertain internal consistency in several steps: First, we examine the face validity of randomly selected, individual patient “traces.” A graphical representation of a patient trace in South Africa is presented in Figure S2, illustrating CD4 cell count, HIV RNA, and clinical events, including TB, over a hypothetical patient’s lifetime. These detailed views of a patient’s month-to-month experience offer both a first check on the reasonableness of the output and a convenient means of debugging. Next, we conduct checks of “internal validity,” verifying that the output of the model accurately reproduces the data used to derive input parameters. Figure S3 illustrates the internal validation of South African data as used to derive critical model input parameters such as monthly mortality and OD incidence rates, stratified by CD4 count [5, 11]. These data are used as model input parameters, and survival curves are generated by the model. The curves are then compared to published survival curves from the original South African cohorts, and are found to closely approximate the clinical data.

**Technical Appendix Figures:**

**Figure S1: ART Scale Up Strategies**

Bubble diagram of the ART scale up strategies toward the 2009 WHO guidelines, as applied to the CEPAC International model. The figure demonstrates the flow of hypothetical patients in the model. Patients begin with a true CD4 cell count and HIV RNA level and traverse along a “natural history” course until they are observed to develop a WHO Stage III or IV disease and/or are observed to become ART-eligible, as defined by the simulated strategy. Once on ART, patients may develop an ART-related toxicity at frequencies depending on the ART components (stavudine vs. tenofovir). If the strategy specifies, a second-line ART regimen may be available. Patients may die from any health state.

**Figure S2: Course of Disease by CD4 cell count and HIV RNA**

A graphical representation of the course of disease for a single, hypothetical patient in South Africa. The figure illustrates CD4 cell count, HIV RNA, and clinical events, including TB, over the patient’s lifetime. These detailed views of a patient’s month-to-month experience offer both a first check on the reasonableness of the output and a convenient means of debugging.

**Figure S3: Validation of South African Natural History Data in CEPAC**

Validation of CEPAC-International (South Africa) natural history and treatment output from reported data from the CTAC, South Africa cohort (Badri et al. AIDS 2004, figure 2).

**Literature Cited**

**Technical Appendix, Table TA 1**

|  | **Non-nucleoside reverse transcriptase inhibitor used in first-line regimen** | **ART initiation threshold** | **Number of available ART regimens** |
| --- | --- | --- | --- |
| 1. | Stavudine | WHO clinical criteria | One-line |
| 2. | Stavudine | CD4 < 200/μl | One-line |
| 3. | Stavudine | CD4 < 350/μl | One-line |
| 4. | Tenofovir | WHO clinical criteria | One-line |
| 5. | Tenofovir | CD4 < 200/μl | One-line |
| 6. | Tenofovir | CD4 < 350/μl | One-line |
| 7. | Stavudine | WHO clinical criteria | Two-lines |
| 8. | Stavudine | CD4 < 200/μl | Two-lines |
| 9. | Stavudine | CD4 < 350/μl | Two-lines |
| 10 | Tenofovir | WHO clinical criteria | Two-lines |
| 11. | Tenofovir | CD4 < 200/μl | Two-lines |
| 12. | Tenofovir | CD4 < 350/μl | Two-lines |

* The cohort begins with an assumed reference strategy of a stavudine-based single line regimen, initiation upon meeting WHO clinical criteria (top row).

**Technical Appendix, Table TA 2**

| **Parameter** | | | | | | | | **Base case value** | | | | | | | | | | | **Reference** |
| --- | --- | --- | --- | --- | --- | --- | --- | --- | --- | --- | --- | --- | --- | --- | --- | --- | --- | --- | --- |
| **Natural history of disease** | | | | | | | | | | | | | | | | | | | |
|  | | | Mean monthly CD4 cell decline by HIV RNA level, cells/µL | | | | | | | | | | | | | | | | [1] |
|  | | |  | | | | > 100,000 – 30,001 copies/ml | 6.4 | | | | | | | | | | |  |
|  |  |  |  | | | | 10,001 – 30,000 copies/ml | 5.4 | | | | | | | | | | |  |
|  |  |  |  | | | | 3,001 – 10,000 copies/ml | 4.6 | | | | | | | | | | |  |
|  |  |  |  | | | | 501 – 3,000 copies/ml | 3.7 | | | | | | | | | | |  |
|  |  |  |  | | | | <500 copies/ml | 3.0 | | | | | | | | | | |  |
|  | | Incidence of HIV-related diseases in the absence of  ART by CD4 count (cells/μL), rate per 100 PY | | | | | | ≤50 | | 51 – 100 | | 101 – 200 | | 201 – 350 | | 350 – 500 | | >500 | [2] |
|  | |  | | | Severe opportunistic diseases | | | |  | |  | |  | |  | |  | |  |
|  | |  | | |  | Bacterial | | 8.49 | | 3.60 | | 3.60 | | 1.57 | | 0.99 | | 0.99 |  |
|  | |  | | |  | Fungal | | 26.91 | | 4.99 | | 4.99 | | 1.21 | | 0.25 | | 0.25 |  |
|  | |  | | |  | Active tuberculosis | | 23.76 | | 13.73 | | 13.73 | | 8.12 | | 2.50 | | 2.50 |  |
|  | |  | | |  | Toxoplasmosis | | 0.71 | | 0.27 | | 0.27 | | 0.00 | | 0.00 | | 0.00 |  |
|  |  | | |  | | Non-tuberculosis mycobacteriosis | | 3.58 | | 0.40 | | 0.40 | | 0.00 | | 0.12 | | 0.12 |  |
|  |  | | |  | | *Pneumocystis jiroveci* pneumonia | | 1.43 | | 0.27 | | 0.27 | | 0.00 | | 0.12 | | 0.12 |  |
|  |  | | |  | | Other WHO stage 4-defining diseases | | 31.29 | | 8.20 | | 8.20 | | 3.57 | | 2.99 | | 2.99 |  |

**Supplementary Appendix, Table A2 (Continued)**

| **Parameter** | | | | | | | **Base case value** | | | | | | **Reference** |
| --- | --- | --- | --- | --- | --- | --- | --- | --- | --- | --- | --- | --- | --- |
|  | | Incidence of HIV-related diseases in the absence  of ART by CD4 count (cells/μL), rate per 100  PY | | | | | ≤50 | 51 – 100 | 101 – 200 | 201 – 350 | 350 – 500 | >500 | [2] |
|  |  | | | Mild opportunistic diseases | | |  |  |  |  |  |  |  |
|  |  | | | | | Fungal muco-cutaneous disease | 42.94 | 24.81 | 24.81 | 15.45 | 7.16 | 7.16 |  |
|  |  | | | | | Other WHO stage II | 37.28 | 32.85 | 32.85 | 37.93 | 30.45 | 30.45 |  |
|  | | Monthly probability of death from HIV-related  disease by CD4 count **(**cells/μL), % | | | | | ≤50 | 51 – 100 | 101 – 200 | 201 – 350 | 350 – 500 | >500 | [2] |
|  | | | Severe opportunistic diseases | | | | | |  |  |  |  |  |
|  | | |  | | Bacterial | | 2.86 | 2.86 | 2.86 | 2.86 | 2.86 | 2.86 |  |
|  | | |  | | Fungal | | 4.21 | 4.21 | 4.21 | 4.21 | 4.21 | 4.21 |  |
|  | | |  | | Active tuberculosis | | 1.78 | 1.78 | 1.78 | 1.78 | 1.78 | 1.78 |  |
|  | | |  | | Toxoplasmosis | | 0.00 | 0.00 | 0.00 | 0.00 | 0.00 | 0.00 |  |
|  | | |  | | Non-tuberculosis mycobacteriosis | | 12.45 | 12.45 | 12.45 | 12.45 | 12.45 | 12.45 |  |
|  | | |  | | *Pneumocystis jiroveci* pneumonia | | 13.32 | 13.32 | 13.32 | 13.32 | 13.32 | 13.32 |  |
|  | | |  | | Other WHO stage 4-defining diseases | | 8.06 | 8.06 | 8.06 | 8.06 | 8.06 | 8.06 |  |

**Supplementary Appendix, Table A2 (Continued)**

| **Parameter** | | | **Base case value** | | | | | | **Reference** |
| --- | --- | --- | --- | --- | --- | --- | --- | --- | --- |
|  | Monthly probability of death from HIV-  related diseases by CD4 count  **(**cells/μL), % | | ≤50 | 51 – 100 | 101 – 200 | 201 – 350 | 350 – 500 | >500 | [2] |
|  | Mild opportunistic diseases | |  |  |  |  |  |  |  |
|  |  | Fungal muco-cutaneous disease | 1.39 | 1.39 | 1.39 | 1.39 | 1.39 | 1.39 |  |
|  |  | Other WHO stage II | 0.40 | 0.40 | 0.40 | 0.40 | 0.40 | 0.40 |  |
