# Supplementary material for: Scaling Up the 2010 World Health Organization HIV Treatment Guidelines in Resource-Limited Settings: A Model-Based Analysis
Source: PLoS Med. 2010 Dec 21;7(12):e1000382. doi: 10.1371/journal.pmed.1000382 (PMC3014084; doi:10.1371/journal.pmed.1000382)
Supplement: Text S2 — WHO priorities; sensitivity analyses addendum, part 1. (0.26 MB DOC) [file pmed.1000382.s006.doc]

**Scaling up the 2010 WHO HIV treatment guidelines in resource-limited settings:**

**A model-based analysis**

**Sensitivity Analyses Addendum 1**

**Walensky et al.**

**This sensitivity analyses addendum provides additional information on the results of multiple sensitivity analyses not included in the main manuscript.**

**Sensitivity Analyses**

This section provides numerous supplementary analyses which shed additional light on primary findings reported in the paper.

The projected survival curves provided in the main paper are provided 1) only for the strategies selected as optimal in Table 2, and 2) for analyses projected for a long-term horizon. In Table SA 1, we provide the 1- through 5-year survival rates for all 12 strategies examined. Furthermore, in Figure S4, we provide the survival curves of the step-wise strategies selected, on a 5-year horizon. From these results illustrated on a short-term horizon, we can appreciate the substantial benefits of earlier ART initiation. However, benefits of second-line therapy and the impact of tenofovir cannot be well-appreciated until later time points, as show in the manuscript.

We selected for the base case a cohort with a mean CD4 cell count of 375/µl to demonstrate the benefit that might be achieved with earlier ART initiation. In Sensitivity Addendum, Table SA 2A-C, we demonstrate the results, should the cohort have a lower CD4 cell count. These clinical benefits of earlier ART initiation, in this case, are diminished, though the cost-effectiveness results remain robust (Table SA 3c1-3). Indeed, in this “sicker” cohort, the benefits seen from all interventions are blunted.

Sensitivity Analyses Addendum, Table SA 3(a & b) provides the details of the cost-effectiveness analyses, noted in the main paper, specifically, those on second-line regimen costs and CD4 monitoring costs. Tables SA 3(a-c) provide detailed results to support the statement in the paper noting that reasonable variations in initial CD4, second-line ART costs, and monitoring costs do not lead to policy-relevant changes to our conclusions.

**Sensitivity Analyses Addendum Figure Legend**

**Figure Legends:**

**Figure S4: Patient Survival in the First Five Years**

Model-projected survival curves (undiscounted) of the reference ART strategy (stavudine/WHO/one-line) and the three strategies projected to maximize life expectancy in step-wise progression toward the 2009 WHO guidelines (see Results and Table 2 for details). This figure illustrates outcomes over the next five years, while figure 2 in the paper demonstrates a lifetime horizon.

**Literature Cited**

1*. Gold MR, Siegel JE, Russell LB, Weinstein MC, ed. Cost-effectiveness in Health and Medicine. 1996, Oxford University Press: New Yo*rk.

**Sensitivity Analyses Addendum, Table SA 1: Model-projected probability of survival for each of the 12 strategies, as well as no ART, on a 1-5 year time horizon.**

|  | **Year 1** | **Year 2** | **Year 3** | **Year 4** | **Year 5** |
| --- | --- | --- | --- | --- | --- |
| **no ART** | 0.94 | 0.88 | 0.71 | 0.51 | 0.27 |
| **Stavudine/WHO/one-line** | 0.96 | 0.92 | 0.83 | 0.74 | 0.65 |
| **Tenofovir/WHO/one-line** | 0.96 | 0.92 | 0.83 | 0.74 | 0.66 |
| **Stavudine/<200/µl/one-line** | 0.96 | 0.92 | 0.87 | 0.84 | 0.80 |
| **Tenofovir/<200/µl/one-line** | 0.96 | 0.92 | 0.87 | 0.85 | 0.82 |
| **Stavudine/<350/µl/one-line** | 0.97 | 0.95 | 0.94 | 0.92 | 0.87 |
| **Tenofovir/<350/µl/one-line** | 0.97 | 0.95 | 0.94 | 0.93 | 0.89 |
| **Stavudine/WHO/two-lines** | 0.96 | 0.92 | 0.83 | 0.74 | 0.66 |
| **Tenofovir/WHO/two-lines** | 0.96 | 0.92 | 0.83 | 0.75 | 0.67 |
| **Stavudine/<200/µl/two-lines** | 0.96 | 0.92 | 0.87 | 0.85 | 0.83 |
| **Tenofovir/<200/µl/two-lines** | 0.96 | 0.92 | 0.87 | 0.85 | 0.83 |
| **Stavudine/<350/µl/two-lines** | 0.97 | 0.95 | 0.94 | 0.92 | 0.91 |
| **Tenofovir/<350/µl/two-lines** | 0.97 | 0.95 | 0.94 | 0.93 | 0.91 |

**Sensitivity Analyses Addendum, Table SA 2a: Projected life expectancies associated with alternative choices in the step-wise progression toward implementation of the new WHO HIV treatment guidelines; sensitivity analysis with mean cohort CD4 cell count of 250/µl, standard deviation of 25/µl.***

|  | **5-year**  **Survival (%)** | **Projected Life Expectancy (months)** | **Δ Projected Life Expectancy (months)*** |
| --- | --- | --- | --- |
| **Step #1: Begin with stavudine/WHO/one-line (reference strategy) (4 options)** | 57 | 83.4 | --- |
| (1) switch from stavudine to tenofovir, or | 61 | 98.0 | 14.6 |
| (2) add CD4 monitoring capacity, initiate ART at CD4 <200/µl, or | 75 | 102.6 | 19.2 |
| (3) add second-line ART regimen, or | 61 | 105.5 | 22.1 |
| **(4) add CD4 monitoring capacity, initiate ART at CD4 <350/µl** | **79** | **108.3** | **24.9** |
| **Step #2: Begin with stavudine/< 350/µl/one-line (2 options)** | 79 | 108.3 | ---  --- |
| (1) switch from stavudine to tenofovir, or | 85 | 129.4 | 21.1 |
| **(2) add second-line ART regimen** | **90** | **161.2** | **52.9** |
| **Step #3: Begin with stavudine/<350/µl/two-lines (1 remaining option)** | 90 | 161.2 | --- |
| **(1) switch from stavudine to tenofovir** | **92** | **177.7** | **16.5** |

*We use the following nomenclature to define the strategies: nucleoside analogue used in first-line/ART initiation criteria/number of available regimens. All strategies with initiation criteria indicated by a CD4 count threshold assume availability of CD4 count monitoring. For each step, the option that maximizes survival is shown in **bold.** The Δ is change compared to the gray-shaded program selected in the previous step.

**Sensitivity Analyses Addendum, Table SA 2b: Projected life expectancies associated with alternative choices in the step-wise progression toward implementation of the new WHO HIV treatment guidelines; sensitivity analysis with mean cohort CD4 cell count of 150/µl, standard deviation of 25/µl.***

|  | **5-year**  **Survival (%)** | **Projected Life Expectancy (months)** | **Δ Projected Life Expectancy (months)*** |
| --- | --- | --- | --- |
| **Step #1: Begin with stavudine/WHO/one-line (reference strategy) (4 options)** | 54 | 74.5 | --- |
| (1) switch from stavudine to tenofovir, or | 61 | 90.4 | 15.9 |
| (2) add CD4 monitoring capacity, initiate ART at CD4 <200/µl, or | 70 | 92.3 | 17.8 |
| (3) add CD4 monitoring capacity, initiate ART at CD4 <350/µl, or | 70 | 92.4 | 17.9 |
| **(4) add second-line ART regimen** | **61** | **95.0** | **20.5** |
| **Step #2: Begin with stavudine/WHO/two-lines (3 options)** | 61 | 95.0 | ---  --- |
| (1) switch from stavudine to tenofovir, or | 65 | 105.0 | 10.0 |
| **(2) add CD4 monitoring capacity, initiate ART at CD4 <200/µl, or** | **86** | **141.0** | **46.0** |
| **(3) add CD4 monitoring capacity, initiate ART at CD4 <350/µl** | **86** | **141.0** | **46.0** |
| **Step #3: Begin with stavudine/<350/µl/two-lines (1 remaining option)** | **86** | 141.0 | --- |
| **(1) switch from stavudine to tenofovir** | **89** | **156.7** | **15.7** |

*We use the following nomenclature to define the strategies: nucleoside analogue used in first-line/ART initiation criteria/number of available regimens. All strategies with initiation criteria indicated by a CD4 count threshold assume availability of CD4 count monitoring. For each step, the option that maximizes survival is shown in **bold.** The Δ is change compared to the gray-shaded program selected in the previous step**.**

**Sensitivity Analyses Addendum, Table SA 2c: Projected life expectancies associated with alternative choices in the step-wise progression toward implementation of the new WHO HIV treatment guidelines; sensitivity analysis with mean cohort CD4 cell count of 100/µl, standard deviation of 25/µl.***

|  | **5-year**  **Survival (%)** | **Projected Life Expectancy (months)** | **Δ Projected Life Expectancy (months)*** |
| --- | --- | --- | --- |
| **Step #1: Begin with stavudine/WHO/one-line (reference strategy) (4 options)** | 51 | 69.3 | --- |
| (1) add CD4 monitoring capacity, initiate ART at CD4 <200/µl, or | 64 | 83.5 | 14.2 |
| (2) add CD4 monitoring capacity, initiate ART at CD4 <350/µl, or | 64 | 83.5 | 14.2 |
| (3) switch from stavudine to tenofovir, or | 59 | 85.2 | 15.9 |
| **(4) add second-line ART regimen** | **59** | **88.0** | **18.7** |
| **Step #2: Begin with stavudine/WHO/two-lines (3 options)** | 59 | 88.0 | ---  --- |
| (1) switch from stavudine to tenofovir, or | 64 | 97.8 | 9.8 |
| **(2) add CD4 monitoring capacity, initiate ART at CD4 <200/µl, or** | **82** | **128.4** | **40.4** |
| **(3) add CD4 monitoring capacity, initiate ART at CD4 <350/µl** | **83** | **128.4** | **40.4** |
| **Step #3: Begin with stavudine/<200/µl/two-lines (3 remaining options)** | **83** | **128.4** | --- |
| (1) initiate ART at CD4 <350/µl, or | 82 | 128.4 | 0.00 |
| **(2) switch from stavudine to tenofovir, initiate ART at CD4 <200/µl, or** | **85** | **142.5** | **14.1** |
| **(3) switch from stavudine to tenofovir, initiate ART at CD4 <350/µl** | **85** | **142.5** | **14.1** |

*We use the following nomenclature to define the strategies: nucleoside analogue used in first-line/ART initiation criteria/number of available regimens. All strategies with initiation criteria indicated by a CD4 count threshold assume availability of CD4 count monitoring. For each step, the option that maximizes survival is shown in **bold.** The Δ is change compared to the gray-shaded program selected in the previous step**.**

**Sensitivity Analyses Addendum, Table SA 3a:** Sensitivity analysis on cost of a second-line regimen (= $100 per person per year [base case $669])

| **Strategy** | **Discounted cost** | **Discounted per person life expectancy**  **months** | **Incremental cost-effectiveness ratio ($/YLS)** |
| --- | --- | --- | --- |
| No ART | 2,540 | 44.9 | -- |
| **Stavudine/<350/µl/one-line** | **5,550** | **104.3** | **610** |
| Stavudine/<200/µl/one-line | 5,740 | 97.3 | Dominated^a^ |
| Tenofovir/<350/µl/one-line | 6,870 | 118.3 | Dominated^b^ |
| Tenofovir/<200/µl/one-line | 6,930 | 109.9 | Dominated^a^ |
| Stavudine/WHO/one-line | 7,440 | 84.5 | Dominated^a^ |
| Tenofovir/WHO/one-line | 8,400 | 93.9 | Dominated^a^ |
| Stavudine/WHO/two-lines | 8,490 | 98.8 | Dominated^a^ |
| Stavudine/<200/µl/two-lines | 8,700 | 126.9 | Dominated^b^ |
| Stavudine/<350/µl/two-lines | 8,980 | 138.8 | Dominated^b^ |
| Tenofovir/WHO/two-lines | 8,990 | 105.2 | Dominated^a^ |
| Tenofovir/<200/µl/two-lines | 9,350 | 135.4 | Dominated^a^ |
| **Tenofovir/<350/µl/two-lines** | **9,690** | **148.2** | **1,130** |

a: indicates strategies that are strongly dominated (more expensive but confer less clinical benefit) [1].

b: indicates strategies that are weakly dominated (result in a higher incremental cost-effectiveness ratio than the next more expensive alternative) [1].

**Sensitivity Analyses Addendum, Table SA 3b: Sensitivity analysis on cost of CD4 test ($75/test [base case $25/test])**

| **Strategy** | **Discounted cost** | **Discounted per person life expectancy**  **months** | **Incremental cost-effectiveness ratio ($/YLS)** |
| --- | --- | --- | --- |
| No ART | 2,590 | 44.9 | -- |
| **Stavudine/<350/µl/one-line** | **5,690** | **104.3** | **630** |
| Stavudine/<200/µl/one-line | 5,960 | 97.2 | Dominated^a^ |
| **Tenofovir/<350/µl/one-line** | **7,010** | **118.3** | **1,140** |
| Tenofovir/<200/µl/one-line | 7,140 | 109.9 | Dominated^a^ |
| Stavudine/WHO/one-line | 7,480 | 84.4 | Dominated^a^ |
| Tenofovir/WHO/one-line | 8,430 | 94.0 | Dominated^a^ |
| Stavudine/WHO/two-lines | 10,190 | 98.8 | Dominated^a^ |
| Tenofovir/WHO/two-lines | 10,700 | 105.0 | Dominated^a^ |
| Stavudine/<200/µl/two-lines | 12,780 | 126.8 | Dominated^b^ |
| Tenofovir/<200/µl/two-lines | 13,330 | 135.4 | Dominated^b^ |
| Stavudine/<350/µl/two-lines | 13,770 | 138.8 | Dominated^b^ |
| **Tenofovir/<350/µl/two-lines** | **14,350** | **148.2** | **2,940** |

a: indicates strategies that are strongly dominated (more expensive but confer less clinical benefit) [1].

b: indicates strategies that are weakly dominated (result in a higher incremental cost-effectiveness ratio than the next more expensive alternative) [1].

**Sensitivity Analyses Addendum, Table SA 3C_1_:** Sensitivity analysis with mean cohort CD4 cell count of 250/µl [standard deviation = 25/µl, base case mean cohort CD4 cell count = 375/µl]

| **Strategy** | **Discounted cost** | **Discounted per person life expectancy**  **months** | **Incremental cost-effectiveness ratio ($/YLS)** |
| --- | --- | --- | --- |
| No ART | 2,250 | 29.7 | -- |
| **Stavudine/<350/µl/one-line** | **5,690** | **92.7** | **660** |
| Stavudine/<200/µl/one-line | 5,810 | 88.0 | Dominated^a^ |
| Stavudine/WHO/one-line | 6,280 | 72.1 | Dominated^a^ |
| **Tenofovir/<350/µl/one-line** | **7,120** | **107.9** | **1,120** |
| Tenofovir/<200/µl/one-line | 7,120 | 102.3 | Dominated^a^ |
| Tenofovir/WHO/one-line | 7,320 | 82.6 | Dominated^a^ |
| Stavudine/WHO/two-lines | 9,130 | 87.1 | Dominated^a^ |
| Tenofovir/WHO/two-lines | 9,690 | 94.1 | Dominated^a^ |
| Stavudine/<200/µl/two-lines | 12,080 | 120.7 | Dominated^b^ |
| Stavudine/<350/µl/two-lines | 12,610 | 128.6 | Dominated^b^ |
| Tenofovir/<200/µl/two-lines | 12,630 | 130.3 | Dominated^b^ |
| **Tenofovir/<350/µl/two-lines** | **13,190** | **139.1** | **2,330** |

a: indicates strategies that are strongly dominated (more expensive but confer less clinical benefit) [1].

b: indicates strategies that are weakly dominated (result in a higher incremental cost-effectiveness ratio than the next more expensive alternative) [1].

**Sensitivity Analyses Addendum, Table SA 3C_2_:** Sensitivity analysis with mean cohort CD4 cell count of 150/µl [standard deviation = 25/µl, base case mean cohort CD4 cell count = 375/µl]

| **Strategy** | **Discounted cost** | **Discounted per person life expectancy**  **months** | **Incremental cost-effectiveness ratio ($/YLS)** |
| --- | --- | --- | --- |
| No ART | 2,500 | 19.7 | -- |
| Stavudine/WHO/one-line | 6,400 | 65.2 | Dominated^b^ |
| Stavudine/<200/µl/one-line | 6,790 | 80.1 | Dominated^b^ |
| **Stavudine/<350/µl/one-line** | **6,790** | **80.2** | **850** |
| Tenofovir/WHO/one-line | 7,690 | 77.1 | Dominated^a^ |
| **Tenofovir/<350/µl/one-line** | **8,450** | **96.0** | **1,260** |
| Tenofovir/<200/µl/one-line | 8,450 | 95.8 | Dominated^a^ |
| Stavudine/WHO/two-lines | 9,220 | 79.6 | Dominated^a^ |
| Tenofovir/WHO/two-lines | 9,890 | 86.9 | Dominated^a^ |
| Stavudine/<200/µl/two-lines | 12,700 | 114.8 | Dominated^b^ |
| Stavudine/<350/µl/two-lines | 12,730 | 115.1 | Dominated^b^ |
| Tenofovir/<200/µl/two-lines | 13,380 | 125.2 | Dominated^b^ |
| **Tenofovir/<350/µl/two-lines** | **13,380** | **125.3** | **2,020** |

a: indicates strategies that are strongly dominated (more expensive but confer less clinical benefit) [1].

b: indicates strategies that are weakly dominated (result in a higher incremental cost-effectiveness ratio than the next more expensive alternative) [1].

**Sensitivity Analyses Addendum, Table SA 3C_3_:** Sensitivity analysis with mean cohort CD4 cell count of 100/µl [standard deviation = 25/µl, base case mean cohort CD4 cell count = 375/µl]

| **Strategy** | **Discounted cost** | **Discounted per person life expectancy**  **months** | **Incremental cost-effectiveness ratio ($/YLS)** |
| --- | --- | --- | --- |
| No ART | 2,310 | 16.1 | -- |
| Stavudine/WHO/one-line | 6,750 | 61.0 | Dominated^b^ |
| **Stavudine/<350/µl/one-line** | **7,390** | **73.1** | **1,070** |
| Stavudine/<200/µl/one-line | 7,400 | 73.1 | Dominated^b^ |
| Tenofovir/WHO/one-line | 8,250 | 73.1 | Dominated^a^ |
| **Tenofovir/<200/µl/one-line** | **9,290** | **88.5** | **1,480** |
| Tenofovir/<350/µl/one-line^c^ | 9,290 | 88.5 | Dominated^a^ |
| Stavudine/WHO/two-lines | 9,370 | 74.5 | Dominated^a^ |
| Tenofovir/WHO/two-lines | 10,160 | 81.9 | Dominated^a^ |
| Stavudine/<200/µl/two-lines | 12,550 | 105.9 | Dominated^b^ |
| Stavudine/<350/µl/two-lines | 12,550 | 106.0 | Dominated^b^ |
| Tenofovir/<350/µl/two-lines | 13,210 | 115.5 | Dominated^b^ |
| **Tenofovir/<200/µl/two-lines** | **13,220** | **115.6** | **1,740** |

a: indicates strategies that are strongly dominated (more expensive but confer less clinical benefit) [1].

b: indicates strategies that are weakly dominated (result in a higher incremental cost-effectiveness ratio than the next more expensive alternative) [1].

c: the cost and clinical outcomes in this strategy are identical to those of Tenofovir/<200/µl/one-line.
